# A biophysically constrained computational model of the action potential of mouse urinary bladder smooth muscle

**DOI:** 10.1371/journal.pone.0200712

**Published:** 2018-07-26

**Authors:** Chitaranjan Mahapatra, Keith L. Brain, Rohit Manchanda

**Affiliations:** 1 Department of Biosciences and Bioengineering, Indian Institute of Technology Bombay, Mumbai, Maharashtra, India; 2 School of Clinical and Experimental Medicine, University of Birmingham, Birmingham, England, United Kingdom; Cinvestav-IPN, MEXICO

## Abstract

Urinary incontinence is associated with enhanced spontaneous phasic contractions of the detrusor smooth muscle (DSM). Although a complete understanding of the etiology of these spontaneous contractions is not yet established, it is suggested that the spontaneously evoked action potentials (sAPs) in DSM cells initiate and modulate the contractions. In order to further our understanding of the ionic mechanisms underlying sAP generation, we present here a biophysically detailed computational model of a single DSM cell. First, we constructed mathematical models for nine ion channels found in DSM cells based on published experimental data: two voltage gated Ca^2+^ ion channels, an hyperpolarization-activated ion channel, two voltage-gated K^+^ ion channels, three Ca^2+^-activated K^+^ ion channels and a non-specific background leak ion channel. The ion channels’ kinetics were characterized in terms of maximal conductances and differential equations based on voltage or calcium-dependent activation and inactivation. All ion channel models were validated by comparing the simulated currents and current-voltage relations with those reported in experimental work. Incorporating these channels, our DSM model is capable of reproducing experimentally recorded spike-type sAPs of varying configurations, ranging from sAPs displaying after-hyperpolarizations to sAPs displaying after-depolarizations. The contributions of the principal ion channels to spike generation and configuration were also investigated as a means of mimicking the effects of selected pharmacological agents on DSM cell excitability. Additionally, the features of propagation of an AP along a length of electrically continuous smooth muscle tissue were investigated. To date, a biophysically detailed computational model does not exist for DSM cells. Our model, constrained heavily by physiological data, provides a powerful tool to investigate the ionic mechanisms underlying the genesis of DSM electrical activity, which can further shed light on certain aspects of urinary bladder function and dysfunction.

## Introduction

In general, urinary incontinence (UI) is defined as the involuntary loss of urine that can be demonstrated objectively and which constitutes a social or hygienic problem [1]. Overactive Bladder or Urge Incontinence is a type of UI, which is associated with a strong premature desire to urinate and correlates with an overactive detrusor smooth muscle (DSM) cell [2]. Spontaneous contractile activity is recorded in DSM strips of mouse, rat, pig, guinea pig and humans, although the number of strips showing activity and the frequency of the contractions varies considerably between species [3, 4, 5]. While the factors regulating the spontaneous contractility are still unclear, three important hypotheses have been advanced as regards primary determinants of bladder dysfunction: (1) the neurogenic hypothesis [6, 7, 8]; (2) the autonomous hypothesis [9]; and (3) the myogenic hypothesis [10, 11].

The pathophysiology of urinary dysfunction is poorly understood. This is in large part because the factors that govern electrical activity and contraction in the muscle of the bladder wall, the detrusor smooth muscle, have not been adequately delineated. A sound understanding of the electrical functioning of DSM cells, as in other excitable cells, rests on the analysis of changes in ionic permeability of the cell membrane. Membrane electrical activity in the form of synaptic potentials and action potentials plays a key role in initiating DSM contraction by mediating influx of Ca^2+^ through voltage-gated Ca^2+^ channels [12, 13, 14, 15, 16, 17, 18, 19]. The ensuing transient elevations in [Ca]_i,_, mediated through multiple subcellular mechanisms, trigger mechanical activity.

One of the puzzling features of DSM electrophysiology is that in any given smooth muscle cell, a considerable variety of action potential (AP, or spike) shapes may be observed [8, 20, 21]. This is in contrast to most other excitable cells, e.g. cardiac myocytes and neurons, where individual cells display a relatively fixed, stereotypical AP under physiological conditions. Unravelling the biophysical features that give rise to the variability observed in DSM may serve to sharpen our understanding of DSM biophysics. Such understanding, however, is impeded by the difficulties inherent in obtaining stable experimental intracellular recordings from smooth muscle cells [22, 23, 24]. Computational models can succinctly capture the often highly nonlinear interactions among various ion channels that participate in generating an action potential and allow the user to investigate the contribution of each ion channel to the overall observed electrical behavior. Over the past decades, computational modeling has been used widely towards these ends for neurons and for cardiac and skeletal muscle cells based on the Hodgkin-Huxley (HH) formulation [25] as well as other formulations such as thermodynamic models and Markov models. Such models have furnished a wealth of insight into the fundamental mechanisms underlying electrical excitability. So far, models for a few smooth muscle cell types, incorporating ionic channels and calcium dynamics, have been developed, such as for intestinal [26], uterine [27, 28, 29], jejunal [30], gastric [31, 32], mesenteric [33], small bowel [34] and arterial [35, 36] smooth muscle cells.

By contrast, computational models for DSM cells are at a relatively nascent stage. Although an electrophysiological model of DSM cell based on the Hodgkin-Huxley formalism was presented recently [37], detailed descriptions of the biophysical characteristics of each of the DSM ionic currents are still lacking. One of the key conflicts between the action potential of the recently published model and the known DSM electrophysiology is that the former is based on an active sodium conductance, whereas most experimental studies do not indicate the presence of voltage-gated sodium ion channels in DSM [23, 38]; other salient conflicts also exist (see Discussion). In addition, information on how these individual ionic currents interplay in order to modulate the shape and time course of the various types of APs recorded in DSM cells is sparse. In this regard, some of the reported effects of pharmacological maneuvers on DSM cell electrophysiology have raised some open biological questions and engendered conflicting hypotheses regarding the precise roles of the K^+^ ion channels in modulating shape of action potentials. For example, according to Li et al., 2017 [39], the elevated conductance of small-conductance calcium-dependent potassium channels (SK) hyperpolarizes the resting membrane potential (RMP) of DSM cells. However, according to Herrera et al., 2002 [40], SK channels modulate the after-hyperpolarization period of the spike in the DSM cells. These conflicts are observed because a particular K^+^ channel blocker may influence the permeability of not just one but two or more of the K^+^ conductances. Thus, such pharmacological manoeuvers do not often suffice to investigate the modulating effects of individual ion channel on spike generation and wave shapes. Here, biophysically detailed computational models can shed light on the in-depth quantitative investigation of DSM cell electrophysiology.

In order to address the aforementioned issues, our primary goal was to develop as robust a computational model of the DSM action potential at the single-cell level as feasible from available experimental data, and from this, both to gain insights into experimental observations made on DSM cells as well as to make predictions regarding the behaviour of these cells under conditions of altered ion channel function. A further goal was to explore the propagation of the computational spike along DSM cells. One of the key properties of spikes is their non-attenuating propagation along lengths of cable-like structures such as axons and muscle cells. Detrusor smooth muscle, like some other smooth muscles, is known to exhibit one-dimensional cable-like behaviour when uniformly polarized at a plane [41]. We thought to ascertain whether our computational action potential would exhibit this property. Towards this end, we constructed a one-dimensional cable model of detrusor smooth muscle by linking five cells end-to-end, the electrical connectivity being provided via gap junctions as described in work performed previously in our laboratory [42]. It is well known, moreover, that when an AP propagates along the length of a cable from a point of initiation, its shape changes. In particular, the convex-upward foot of the AP observed at the point of initiation turns into a concave-upward foot at a distance greater than a few space constants. This is because the convex foot, a passive depolarization induced by external current injection or by synaptic input, fades with distance and is replaced, for the purpose of regenerating the AP along the cable, by local circuit currents which give rise to a concave foot. We also set out to see whether this prediction would be obeyed by our action potential, as this would further bolster the robustness of our model. We extended our investigation by inducing the AP in a 1-D strand model of DSM tissue to study the effects of intercellular gap junction resistance on AP propagation.

Towards developing a model constrained by biophysical data, we have (i) clearly cited the sources of data used to derive model parameters, (ii) described how optimal working values for parameters were arrived at, and (iii) stated whether parameters were borrowed directly from experimental data or modified in order to secure an acceptable match between simulated and experimental APs. We proceeded to validate the simulated ionic currents in DSM cells against the currents recorded experimentally. The known complement of ion channels was integrated to generate the most commonly observed spike-type action potential recorded in DSM cells (see Methods, Discussion). We elicited APs by both external current injection and physiologically realistic inputs represented by synaptic potentials, the APs subsequently being verified against the experimentally recorded signals. Our validated model was then employed in order to resolve existing conflicts, and to gain new biological insights that are experimentally testable (See Discussion). Some preliminary results of this investigation have previously been communicated in brief [43, 44, 45, 46].

## Methods

### Model development

We have considered cylindrical single cell morphology for our model where the cylinder length and diameter values (Table 1) are chosen to represent a DSM cell. Table 1 also provides membrane capacitance (C_m_), membrane resistance (R_m_) and axial resistance values for our model. The individual membrane current components that were modeled were (i) three inward currents: L-type and T-type Ca^2+^ currents (I_CaL_ and I_CaT_), a hyperpolarization-activated current (I_h_); (ii) six outward K^+^ currents: two voltage-gated K^+^ currents (I_Kv1_, I_KCNQ_), an ATP-dependent K^+^ current (I_KATP_) and three Ca^2+^-activated K^+^ currents (I_BK(Ca)_, I_SK(Ca)_, I_IK(Ca)_); and (iii) an outward back-ground leak current (I_Leak_).

**Table 1:** Values of model parameters.

Quantity	Experimental	Reference
Cell Length	200 μm	Fry et al. (1999) [41]
Cell Diameter	6 μm	Fry et al. (1999) [41]
Membrane Resistivity	138 kΩ.cm^2^	Fry et al. (1999) [41]
Cytoplasmic Resistivity	183 Ω.cm	Fry et al. (1999) [41]
Specific Membrane Capacitance	1 μF/cm^2^	Standard Value

To generate a calcium transient, this model also incorporates a simple calcium dynamics based on exponential function (details in last section of method). Formulation of a conceptual model, which is expressed in a mathematical form, is the first step in translation of a physical system to a computational model. Here the conceptual model for individual ionic current is based on the classical Hodgkin-Huxley approach [47]. The cell membrane is described as an equivalent electrical circuit consisting of a membrane capacitance connected in parallel with a number of variable conductances representing the ion channels.

**Fig 1:**
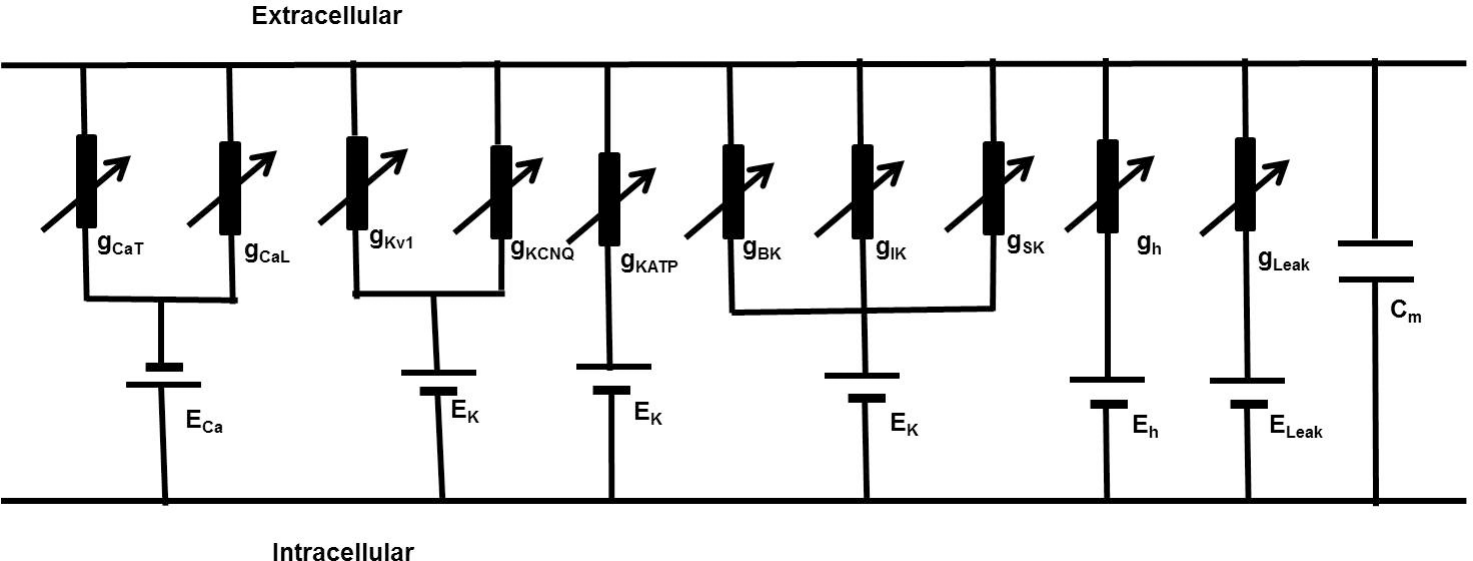
A DSM cell parallel conductance model. It consists of voltage gated *Ca*^2+^ channels, Voltage gated *K*^+^ channels, *Ca*^2+^ activated *K*^+^ channels and leakage currents. Applying Kirchhoff’s current law after injecting stimulus current I_stim_, we get the following differential equation describing changes in transmembrane potential *V*_*m*_.

Fig 1 presents a parallel conductance model consisting of a *C*_*m*_ shunted by a variety of ion channel conductances g_ion_ with respective Nernst potential E_ion_.

$$\frac{dV_{m}}{dt}=-\frac{1}{C_{m}}\;(I_{Ca}+I_{K}+I_{h}+I_{leak}+I_{stim})$$ (1)

All membrane currents except large conductance (BK) Ca^2+^-dependent activation *K*^+^ channel were modeled using the Hodgkin-Huxley formalism:

$$I=\bar{g}{\left[m(V_{m},t,{\left[{Ca}^{2+}\right]}_{i})\right]}^{x}{h[(V_{m},t,{\left[{Ca}^{2+}\right]}_{i})]}^{y}(V_{m}-E_{rev})$$ (2)

where $\bar{g}$ is maximum ionic conductance, E_rev_ is the ion’s reversal potential, the dimensionless gating variable ‘m’ describes the time/voltage/Ca^2+^-dependent activation and ‘h’ is the time/voltage/Ca^2+^-dependent inactivation of the channel conductance. The ‘x’ and ‘y’ are power to the functions.

The variation of each gating variable (m or h) can be expressed by first order differential Eqs (3 and 4)

$$\frac{dm(V_{m},t)}{dt}=\frac{m_{\infty }(V_{m})-m(V_{m},t)}{\tau_{m}}$$ (3)

$$\frac{dh(V_{m},t)}{dt}=\frac{h_{\infty }(V_{m})-h(V_{m},t)}{\tau_{h}}$$ (4)

where m_∞_ and h_∞_ are the steady-state values, τ_m_ and τ_h_ the time constants, all being functions of voltage and/or intracellular Ca^2+^ ionic concentrations.

Here the state parameter dependence on v_m_ for ion channels is described by the Boltzman equation

m∞(Vm,t)=11+exp((Vm+Vm12)/Sm) (5)

h∞(Vm,t)=11+exp((Vm+Vh12)/Sh) (6)

Where V_1/2_ is the half activation potential and S is the slope factor.

BK channels kinetics have been described by a 10-state Markov model (MM) according to a model developed by Cox et al., 1997 [48] and Cox 2014 [49] in which the channel’s Ca^2+^-dependence is modelled at a finer grain, thus affording greater accuracy on this front than the HH formalism. We therefore adapted this multi-state Markov model for the BK conductance. In this model there are five closed “horizontal” conformation states, namely C0, C1, C2, C3 and C4. Similarly, there are five open-oriented “horizontal” conformation states O0, O1, O2, O3 and O4, each corresponding to the appropriate closed state. The MM topology description includes the cooperative Ca^2+^ binding among the states to illustrate the interaction of four alpha subunits of the BK channel. The horizontal transitions among the closed states (C0, C1, C2, C3, C4) and among the open states (O0, O1, O2, O3, O4) are Ca^2+^ dependent. In comparison, vertical transitions occur between corresponding pairs of closed and open states (i.e., C0—O0, C1—O1, C2—O2, C3—O3) and are voltage dependent. A special case is constituted by the vertical transitions between C4 and O4, which are both voltage and Ca^2+^ dependent. All parameters and their values are given in the supplementary document. The open conformation state O4 permits the flow of K^+^ ions through the BK channels under the instantaneous electrochemical driving force (EDF). The BK current, I_BK_ is calculated by the following equation

$$I_{BK}=\bar{g_{BK}}*O*(V-E_{K})$$ (7)

where $\bar{g_{BK}}$ is the maximum conductance and O is summation of O1, O2, O3 and O4.

### Calcium dynamics

In order to describe the calcium-dependent gating of Ca^2+^-dependent potassium channels and to update the equilibrium potential of the Ca^2+^ ion, it was necessary to calculate the intracellular Ca^2+^ concentration. Since the parameters governing many important factors required in order to describe intracellular Ca^2+^ handling are not known, including its diffusion, buffering, release, Na^+^-Ca^2+^ exchanger, and pump extrusion, we did not incorporate a biophysically detailed realistic intracellular Ca^2+^ dynamics in our model. Instead, we assumed that the Ca^2+^ which enters via Ca^2+^ channels instantaneously diffused within a thin sub-membrane shell and that determining the decay of [Ca^2+^]_i_ could be lumped into a single-exponential function [50].

$$\frac{d{\left[{Ca}^{2+}\right]}_{i}}{dt}=-(1000*\frac{i_{Ca}}{2*F*d})-(\frac{{\left[{Ca}^{2+}\right]}_{i\infty }-{\left[{Ca}^{2+}\right]}_{i}}{\tau_{r}})$$ (8)

where, i_Ca_ is the inward Ca^2+^ flux due to voltage gated Ca^2+^ channels, d is the depth of the sub-membrane shell, [Ca^2+^]_i∞_ is the baseline Ca^2+^ concentration, F is the Faraday’s constant and τ_r_ is the time constant.

### Models of synaptic inputs

The simplest model used for synaptic input assumes an instantaneous rise of the synaptic conductance g_syn_(t) from 0 to maximum conductance ${\bar{g}}_{\mathrm{syn}}$ at time instant t_0_ followed by an exponential decay with a time constant τ:

$$g_{syn}(t)={\bar{g}}_{syn}e^{\frac{-(t-t_{0})}{\tau }}$$ (9)

Another popular synaptic model, the “alpha function” [25], describes a conductance that has a rising phase with finite rise time

$$g_{\mathrm{syn}}\left(t\right)={\bar{g}}_{\mathrm{syn}}\frac{t-t_{0}}{\tau }e{(}^{1-\frac{\left(t-t_{0}\right)}{\tau }})$$ (10)

However, due to a single time constant, τ, the time courses of the rise and decay are correlated and cannot be set independently. So for a physiologically realistic model, we have used a more general function describing synaptic conductance profiles consisting of a sum of two exponentials, one generating the rising and one generating the decay phase [51]. It allows these time constants to be set independently such that τ _rise_ ≠ τ _decay_, and for t ≥ t_0_

$$g_{syn}(t)={\bar{g}}_{syn}f(e^{\frac{-(t-t_{0})}{\tau_{decay}}}-e^{\frac{-(t-t_{0})}{\tau_{rise}}})$$ (11)

The normalization factor f is included to ensure that the amplitude equals ${\bar{g}}_{\mathrm{syn}}$.

$$f=\frac{1}{{-e}^{\frac{-(t-t_{0})}{\tau_{rise}}}+e^{\frac{-(t-t_{0})}{\tau_{decay}}}}$$ (12)

### One-dimensional strand model for spike propagation

As outlined in the Introduction, to further test the robustness of our AP model, we ascertained whether our computational AP, validated at the single-cell level, would successfully propagate in a cable-like structure. In order to accomplish this, we started by setting up the action potential in a single cell elongated to a large length (22.2 mm). We divided the elongated cell into 111 interconnected compartments to behave as a continuous cable [42, 25], where each compartment was spatially isopotential (see Results III). The synaptic stimulus was injected at the midpoint of the cell, x = 11.1 mm and electrical activity was recorded at the point of stimulation, i.e., 11.1 mm (designated R0), and at various distances from the point of stimulation (R2), in order to characterize AP propagation. In syncytial tissues such as smooth muscle, intercellular gap junctions subserve cell-to-cell electrical communication [22, 52]. We therefore extended our model to investigate the effect of gap junction properties on propagated APs in DSM cells. Towards this end we first built a 3-cell model of electrically connected cells, incorporating a gap junction resistance, r_j_, between adjacent cells (see Results III), the resistance r_j_ allowing passage of localized currents by means of point processes mechanisms.

### Model simulations

Action potentials were induced in our DSM cell model by applying either an external stimulus current (I_st_) or a current based on synaptic input (I_syn_). External stimulus current was applied either as a brief rectangular pulse for single AP or with a long rectangular pulses for a series of APs. As the voltage clamp method eliminates the capacitive current, ionic currents can be studied separately. All the equations, symbols and constant parameters are defined in Supporting Information S1 Appendix, S1 Table and S2 Table. Simulations were computed using a fixed time step of 0.02 ms, using Euler Method, in a PC with an Intel (R) Core (TM) i5 CPU with 3.20 GHz dual core processor. The simulation environment used for this model is NEURON [25] used widely for realistic modelling of excitable cells.

The simulation environment used for this model is NEURON [25], employed widely for realistic modelling of excitable cells at both individual and network level in computationally efficient ways. This flexible and powerful simulator creates a virtual platform to simulate a diverse range of electrophysiological activities. In NEURON, cell morphology is modelled via the use of individual sections and compartments. Membrane mechanisms are incorporated via point and distributed process. Two primary scripts in NEURON are HOC and NMODL, which enable the modelling of cell morphology (single DSM cell, long cable and 1-D network), point process mechanisms (current clamp, alpha synapse, and gap junctions), and distributed mechanisms (ion channels and calcium dynamics) respectively.

Our DSM model contains a large number of parameters that must be assigned values based on the available data. Here, the majority of the parameter values have been assigned based on experimental studies. However, a limited number of free parameters, most of which are scaling factors, such as the maximum conductance values for each ionic current are modified to obtain acceptable fits to (i) ionic currents recorded under voltage clamp condition and (ii) action potentials in DSM cells.

This mechanism was designed to be run at a single operating temperature 37 deg C which can be specified by the hoc assignment statement. This mechanism is also intended to be used at other temperatures, or to investigate the effects of temperature changes.

The temperature sensitivity parameter “tadj” is defined as

$$tadj=2^{\frac{\left(celsius-37\right)}{10}}$$ (13)

where Celsius is the "operating temperature".

Having developed the model, we tested its robustness to intrinsic parameter variation. We did this by varying gmax ($\bar{g}$) of each of the ionic conductances stepwise over a range of +/- 20% of its control (default) value. We observed that the simulated AP was robust to changes of this order. Thus, while AP parameters varied in the expected direction for each of the imposed changes of conductance (for instance, elevating the $\bar{g}$ of the CaL resulted in elevation of AP peak amplitude, and vice versa), the AP did not “break down” under the imposition of any of these variations, i.e. it did not undergo any pathological variations in amplitude or wave shape parameters. Similar observations were obtained for variations in other intrinsic parameters such as time constants of the conductances, demonstrating the robustness of our simulated AP.

### Goodness-of-fit measure

Standard error of regression (S) or root mean squared error (RMSE) is a goodness-of-fit measure we used for our fits of simulated action potentials as R^2^ has been found to be unsuitable for such nonlinear comparisons [53]. S is calculated by the following formula:

$$S=\sqrt{\frac{\Sigma {\left(Y_{Expt}-Y_{Sim}\right)}^{2}}{N-K}}$$ (14)

where Y_expt_ is the experimental value, Y_Sim_ is the corresponding fit value from simulation, K = number of parameters used in the fit equation (also known as the degrees of freedom), N is the number of data points. A lower value of S denotes smaller average errors and represents a good fit. We chose a value of 5% of the difference between maximum and minimum values taken by the experimental data as our threshold for a good model. S below this value is considered a good fit.

## Results I: Modeling of DSM cell ion channels

### Voltage gated calcium channels

**Fig 2:**
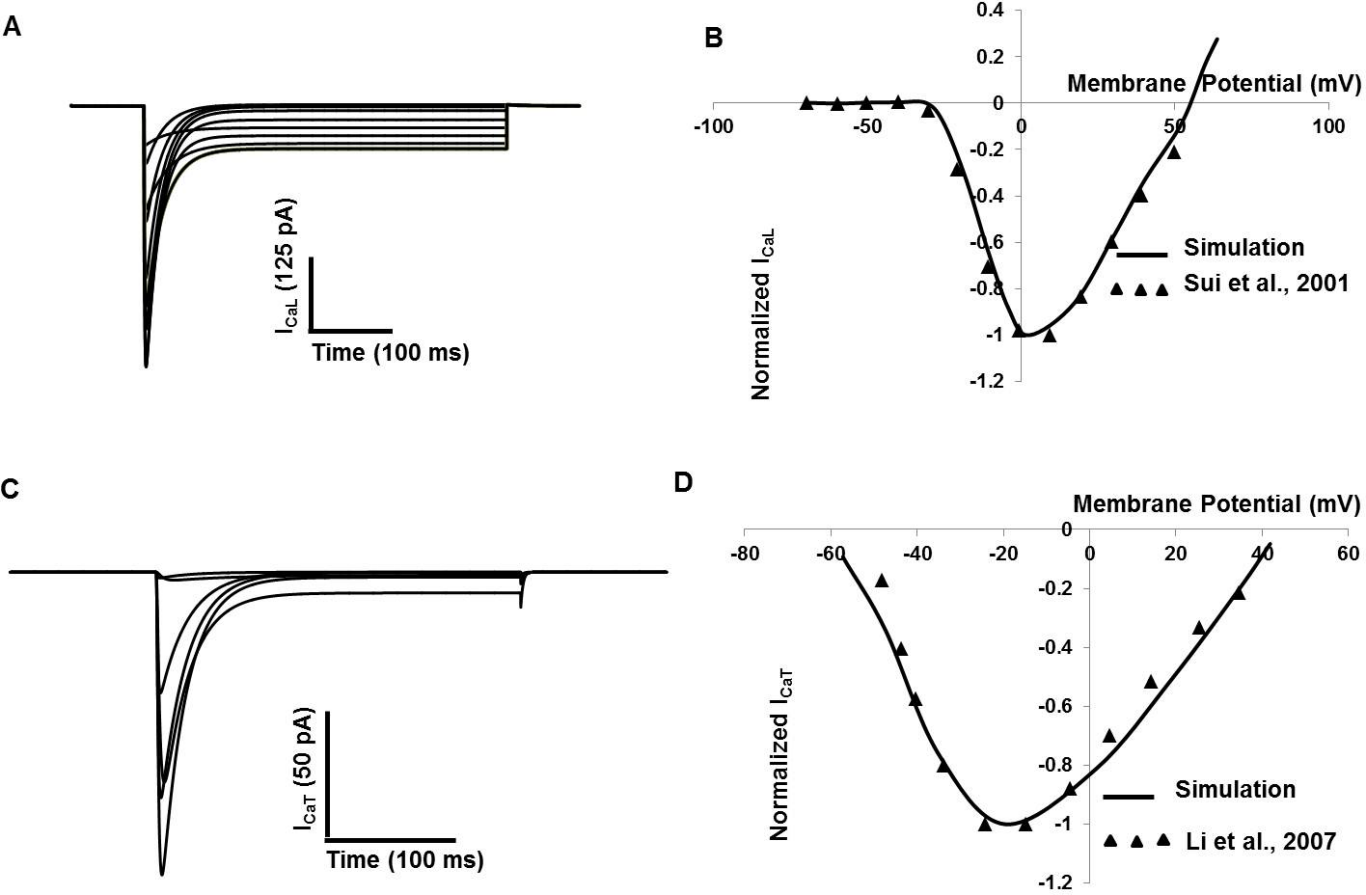
Detrusor smooth muscle I_CaL_ and I_CaT_ model. (A) The holding potential (V_h_) was set at ─90 mV, and the test potentials increased from ─70 mV to 50 mV in 10 mV steps to obtain the I_CaL_ current amplitude (B) Simulated (solid line) normalized I–V relationship of I_CaL_ and experimental (filled triangle) I–V data. (C) The holding potential (V_h_) was set at ─90 mV,and the test potentials stepped from ─70 mV to 50 mV in 20 mV steps to obtain the I_CaT_ current amplitude. (D) Simulated (solid line) normalized I–V relationship of I_CaT_ and experimental (filled triangle) I–V data [56].

Two types of voltage-gated calcium channels (T- and L-type Ca^2+^) channels have been found in DSM cells [16, 17, 18, 19, 54, 55, 56, 57]. L-type Ca^2+^ channels are major contributors of inward current and intracellular ca^2+^ elevation in DSM cell [16, 17, 55]. All biophysical parameters for L-type Ca^2+^ channels used in this model are adapted from [17]. Fig 2A shows simulated voltage-clamp traces of I_CaL_ clamp potentials ranging from ─70 to +50 mV from a holding potential of ─90 mV. In Fig 2B, the simulated (solid line) normalized current-voltage relationship curve for I_CaL_ channel is shown. Experimental data [17] are superimposed (filled triangle).

T-type Ca^2+^ channels are low-voltage activated, with fast activation and rapid inactivation in response to depolarization, and are important in regulating the cell’s excitability. The biophysical and pharmacological properties of T-type Ca^2+^ channels in DSM cells have been documented [12, 18, 54, 56, 57]. All biophysical parameters for T-type Ca^2+^ channel used in our model are adapted from [56]. Fig 2C shows simulated voltage-clamp traces of I_CaT_ at voltage steps of ─70 to +50 mV from a holding potential of ─90mV. In Fig 2D, the simulated (solid line) normalized current-voltage relationship curve for I_CaT_ channel is shown. Experimental data [56] are superimposed (filled triangle).

### Voltage gated potassium channels

**Fig 3:**
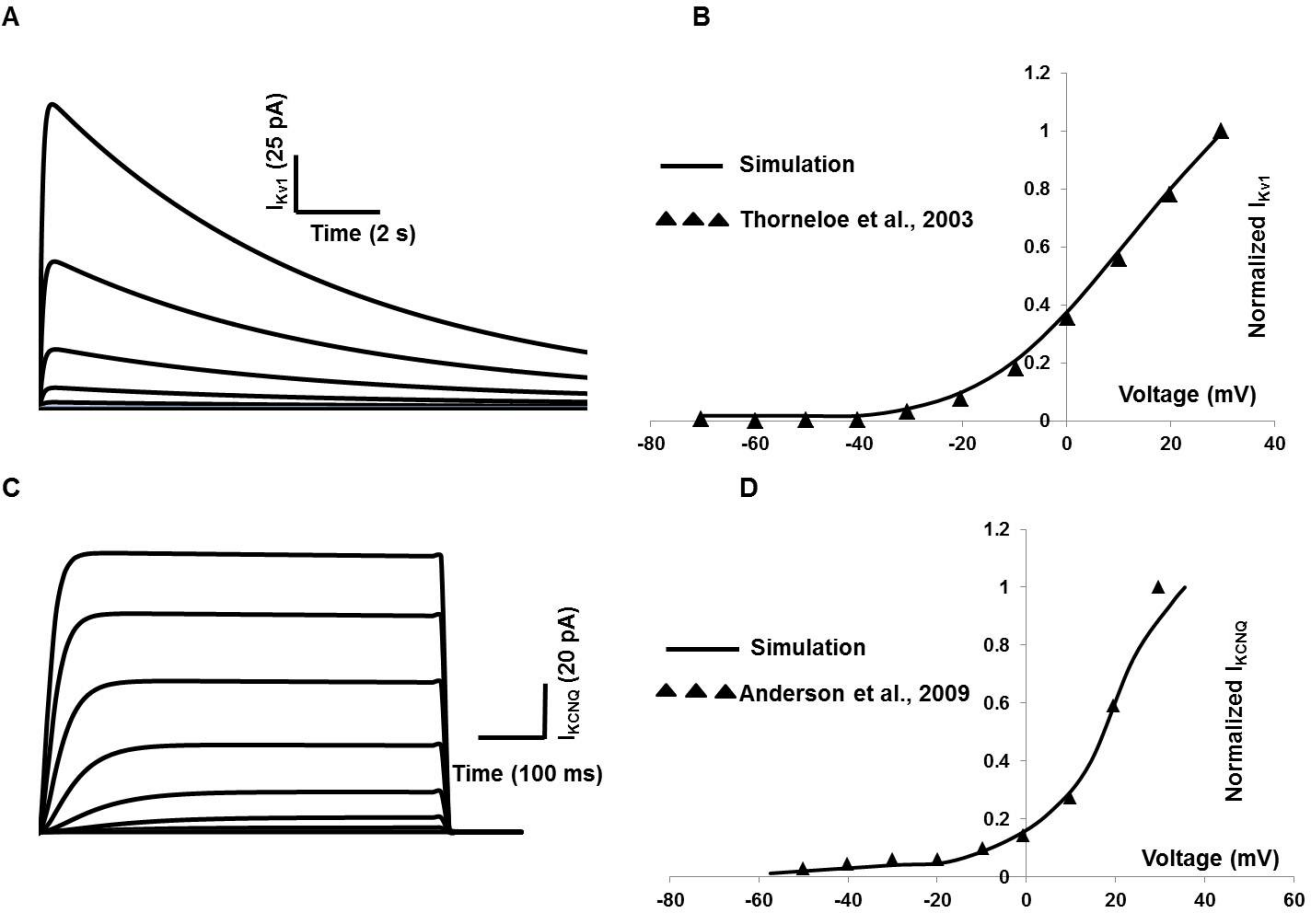
Detrusor I_Kv1_ and I_Kv7_ (KCNQ) model. (A) Inactivation of I_Kv1_ is illustrated in whole-cell currents elicited by 15s depolarizing pulses from a holding potential of ─80 mV to potentials between ─120 and +10 mV. (B) Normalized I_Kv1_ current- voltage curve (solid line from simulation and experimental data in filled triangle from [61] of I_Kv1_. (C) I_Kv7_ (KCNQ) whole-cell currents elicited by 500 ms depolarizing pulses from a holding potential of ─80 mV to potentials between ─80 and +40 mV. (D) Normalized I_Kv7_ current- voltage curve (solid line from simulation and experimental data in filled triangle from [64]).

At least two different types of K_V_ with delayed rectifying properties were found in DSM cells of different animals [20, 23, 58, 59, 60], their dynamics were very slow compared to membrane Ca^2+^ currents in DSM cells. The I_KV1_ has a number of unique characteristics that suggest roles in regulating the resting membrane potential, action potential repolarization and after-hyperpolarization [20, 59, 60]. Modeling parameters for steady-state activation and inactivation are adapted from [61]. Fig 3A shows the inactivation property of I_Kv1_ evoked by a depolarizing pulse lasting for 15 seconds from a holding potential of ─80 mV to potentials between ─120 and +10 mV. The solid line in Fig 3B represents the simulated normalized current-voltage curve, with superimposed (filled triangle) experimental data [61].

KCNQ (also known as Kv7) currents are outwardly delayed rectifying, voltage dependent K^+^ currents that activate at potentials positive to −60 mV and show negligible inactivation [59]. Emerging evidence for expression of KCNQ channels in isolated DSM cells has been addressed by several research groups [62, 63, 64].

The KCNQ currents comprised a component of the total outward current in DSM cells and its modulating role in bladder overactivity is also mentioned in various experimental papers [65, 66]. The pharmacological and biophysical properties of the DSM KCNQ channels are reported in [64]. However, some parameters are not described quantitatively. The similar KCNQ channel is biophysically described in [67] for murine portal vein smooth muscle cells, from where the modeling parameters, namely steady-state activation and inactivation are adopted. Fig 3C represents whole-cell currents evoked by stepping from −80 mV to +40 mV for 500 ms with a holding potential of ─80 mV. Fig 3D shows the normalized current density–voltage graph of KCNQ channel. Experimental data [64] in filled triangle are superimposed against simulation (solid line).

### Calcium-dependent potassium channels

Calcium-activated potassium currents have been suggested to play important roles in suppressing the excitability of DSM cells. There appear to be three calcium-dependent potassium conductances in the DSM cells, the BK (large) conductance, the IK (Intermediate) conductance and the SK (small) conductance [20, 59, 68, 69, 70, 71, 72, 73, 74]. BK channels have a high unitary conductance and are highly voltage- and calcium-sensitive, while IK and SK channels have a lower single-channel conductance, are poorly voltage sensitive or voltage insensitive, but are highly calcium-sensitive [75]. The large conductance Ca^2+^ and voltage activated K^+^ channels (BK channels) are found in the detrusor smooth muscles of several species [15, 75, 76, 77, 78, 79, 80].

**Fig 4:**
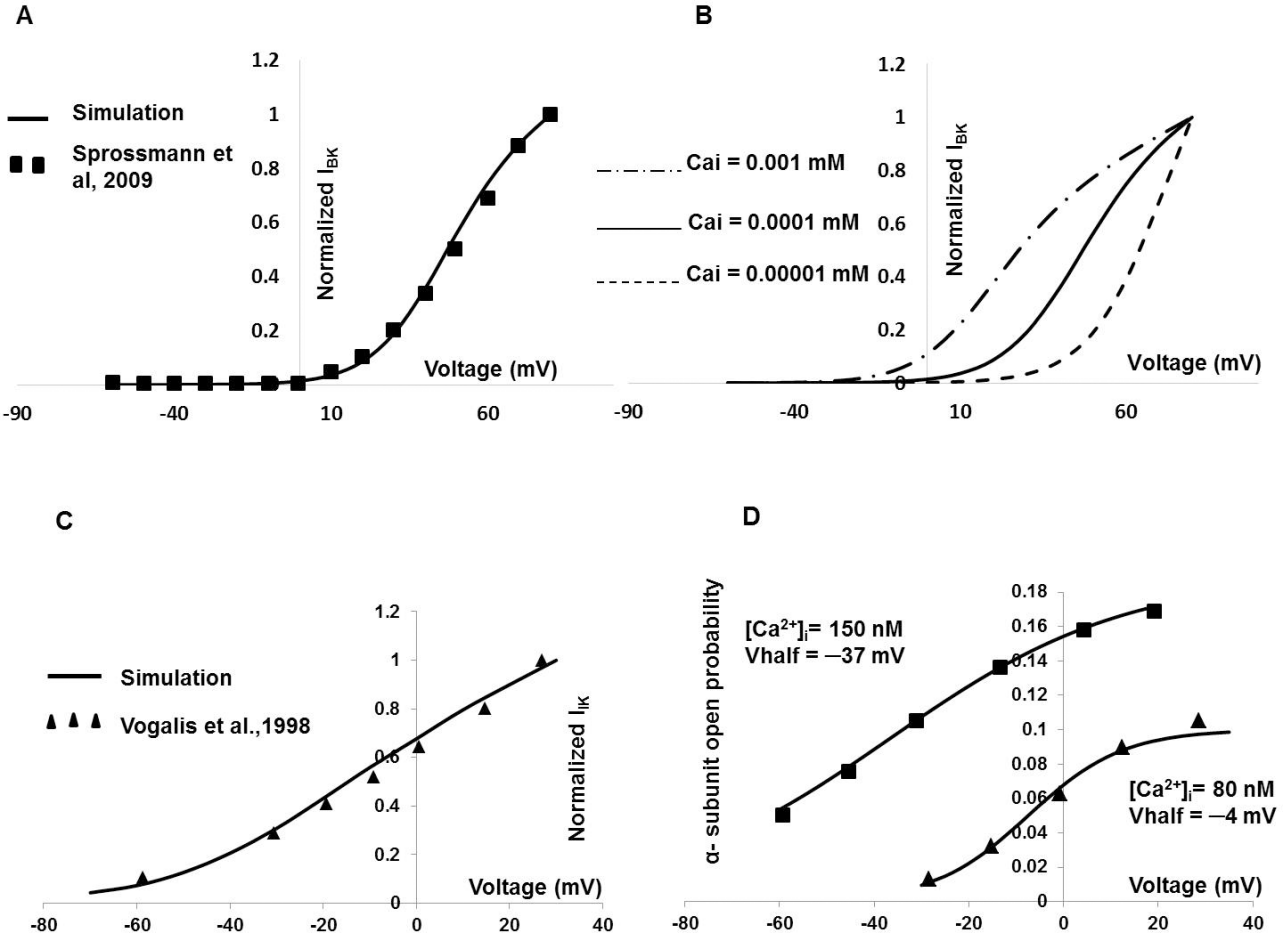
DSM I_BK_ and I_IK_ model. (A) DSM cell I_BK_ model. Fig 4A represents the normalized simulated current-voltage curve (solid line), where experimental data from murine DSM cell [80] are superimposed in filled square. Fig 4B represents the effects of intracellular Ca^2+^ concentration on shifting the current-voltage curve. The [Ca^2+^]_i_ is varied from the control value of 0.0001 mM (solid line) to 0.00001 mM (dashed line) and 0.001 mM (dot and dash line). (C) The solid line represents the normalized simulated I_IK_ current-voltage curve, where experimental data from mouse intestinal cell [81] are superimposed in filled triangle. (D) It represents open probability of α-subunits with respect to varying [Ca^2+^]_i_ in I_IK_ model.

Electrophysiological recordings by Sprossmann et al., 2009 [80] have provided the current-voltage relationship for the BK conductance in murine tissue. Our model’s parameter values were tuned to generate the similar current-voltage relationship under the voltage clamp protocol. In Fig 4A the solid line represents the normalized simulated I_BK_ current-voltage curve, while experimental data from murine DSM cells [80] are superimposed (filled squares). Fig 4B shows the effects intracellular Ca^2+^ on shifting the I-V curve. The current-voltage curve is shifted progressively to the left at higher values of intracellular [Ca^2+^]_i_, as is to be expected. The submembrane Ca^2+^ transient (recorded at a depth of 0.1 μM), which is responsible for the activation of the BK channels, is shown in Figure A in S1 File.

Studies using charybdotoxin suggest that I_IK_ channels may have a functional role in mouse DSM [20]. For I_IK_ channel modeling, the parameters are borrowed from mouse intestinal smooth muscle [81]. The solid line in Fig 4C represents the normalized simulated I_IK_ current-voltage curve, and experimental data from mouse intestinal cell [81] are superimposed (filled triangle). Fig 4D, represents voltage-dependence of the steady state activation of I_IK_ channels in the presence of 80 nM and 150 nM [Ca^2+^]_i_. The filled squares and triangles are experimental data [81] superimposed against simulation (solid line).

**Fig 5:**
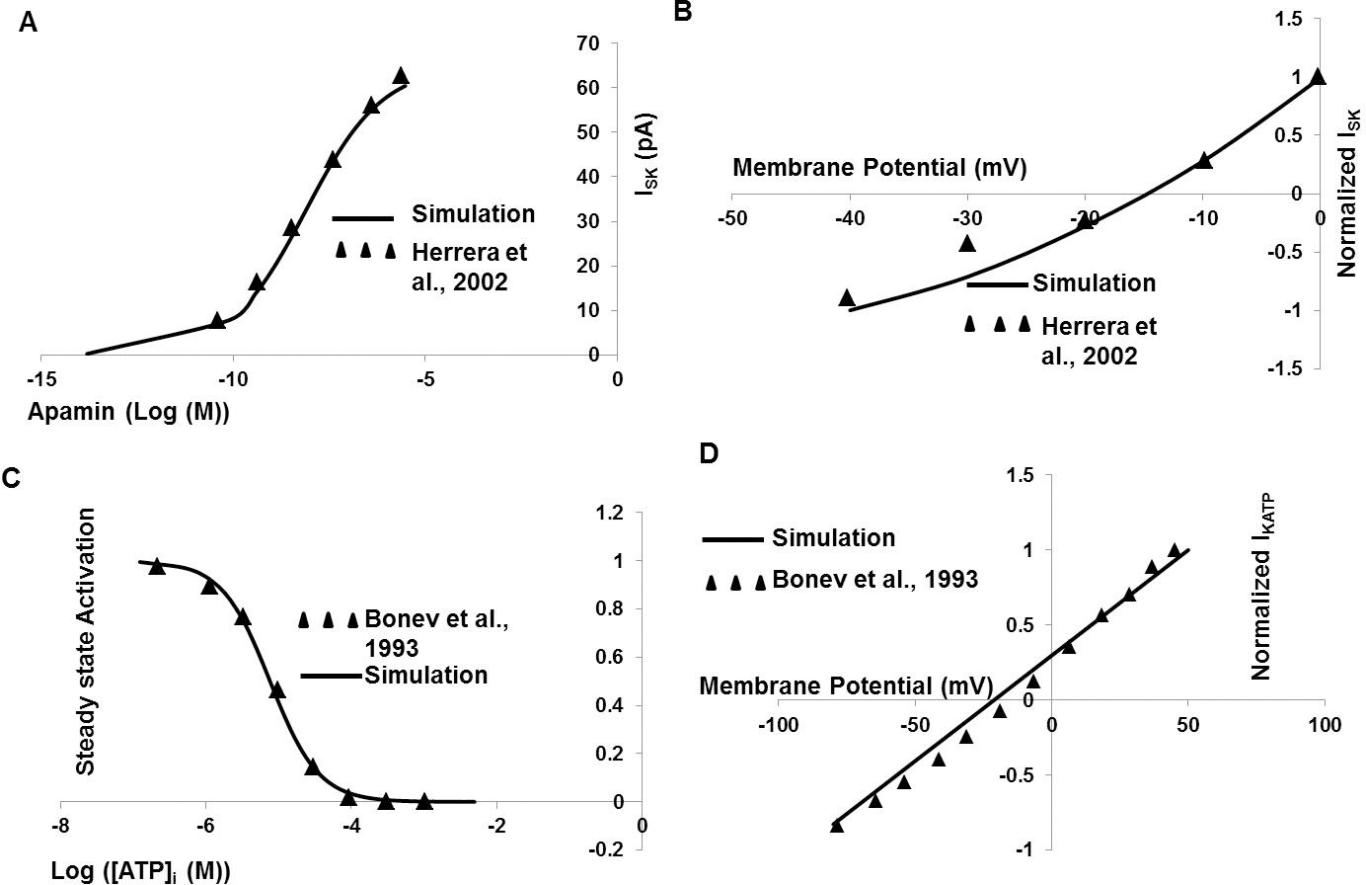
DSM I_SK_ and K_ATP_ model. (A) The normalized I_SK_ current with respect to Apamin in I_SK_ model. (B) The solid line represents the normalized simulated I_SK_ current-voltage curve, where experimental data from murine DSM cell [40] are superimposed (filled triangle). (C) The ATP dependent steady state activation parameter for K_ATP_ channel model. (D) The normalized K_ATP_ current-voltage relationship curve. The solid line represents result from our simulation where filled triangles are superimposed data from experiment [90].

SK channels have a more dominant role in regulating DSM excitability, such that apamin abolishes the fast hyperpolarizations [20, 39, 82, 83, 84, 85]. For I_SK_ channel modeling, the parameters are adopted from guinea-pig urinary bladder smooth muscle [40]. Fig 5A represents normalized I_SK_ current with respect to apamin in I_SK_ model. The solid line in Fig 5B represents the normalized simulated I_SK_ current-voltage curve, while experimental data from murine DSM cell [40] are superimposed (filled triangle).

### ATP sensitive potassium channel

Spontaneous contractions in DSM cells were not affected by K_ATP_ channel blocker drug glibenclamide, but were reduced when K_ATP_ channel opener pinacidil concentrations exceeded 10^−5^ M [86]. Under standard physiological intracellular ATP concentration the K_ATP_ channels are in a closed state and open as ATP concentration falls [59, 87, 88, 89]. Fig 5C represents ATP dependent steady state activation parameter for K_ATP_ channels model, where Fig 5D represents normalized K_ATP_ current-voltage relationship curve. The solid line represents result from our simulation where filled triangle data are fitted from experiment [90].

### Inwardly-rectifying channel

**Fig 6:**
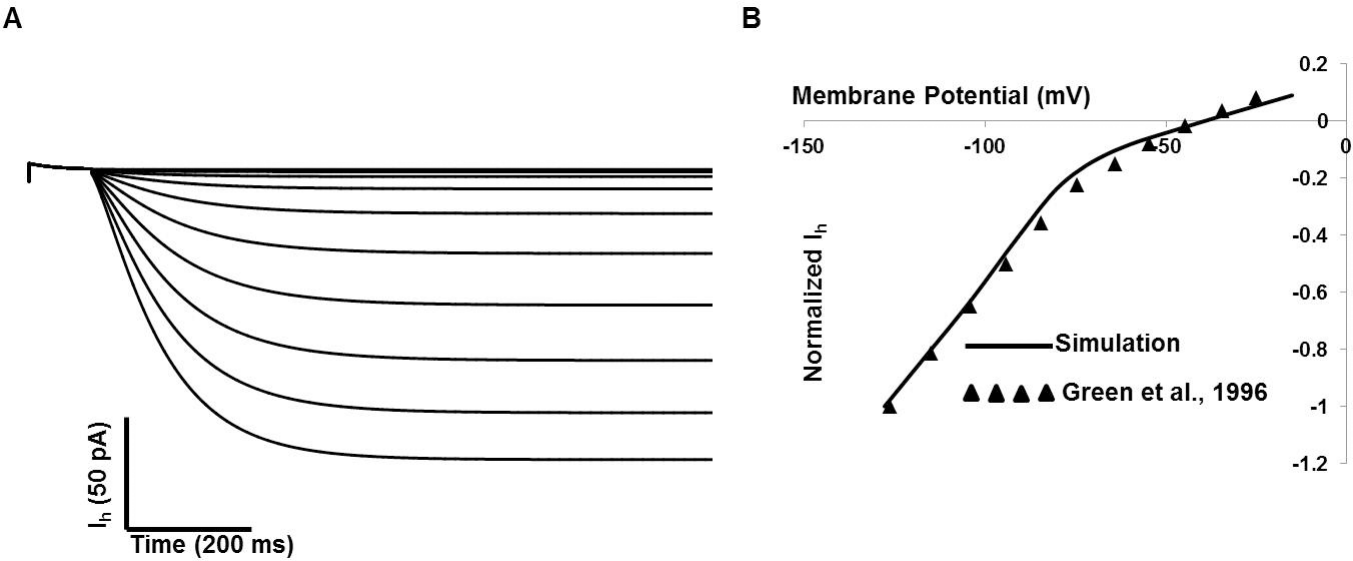
DSM cell I_h_ current model. (A) The simulated current due to voltage-clamp method: voltage steps of ─140 to ─20 mV from a holding potential of ─10mV. (B) The simulated normalized current-voltage relationship curve (solid line) with superimposed experimental data (filled triangle) from [91].

The presence of inward rectifying current in detrusor smooth muscle has been reported mentioned in several reports [23, 59, 91]. This current closely resembles the hyperpolarization-activated current, I_h_, previously described in the other smooth muscles. Our inward rectifying (I_h_) channel model is based on biophysical parameters mentioned in [91]. Fig 6 represents the DSM cell I_h_ current model, Fig 6A illustrates the simulated voltage-clamp of I_IR_ at voltage steps of ─140 to ─20 mV from a holding potential of ─10mV. Fig 6B shows the simulated normalized current-voltage relationship curve in solid line. The experimental data [91] are superimposed [filled triangle].

## Results II–Electrophysiological response

### Resting conditions

The resting membrane potential V_m_ is determined mostly by the balance between depolarizing and repolarizing currents through K_Ca_, K_v,_ K_ATP_, K_leak_ and T–type Ca^2+^ channels. In our model, resting V_m_ is tuned to ─50 mV [8, 20, 21] by adjusting the conductances of all ion channels present, within their respective physiological ranges. Table 2 lists magnitude of ionic conducances and reversal potentials for the ion channels incorporated in our model. Resting intracellular calcium concentration is taken as 150 nM.

**Table 2:** Ion channel paremeters in generating AP.

Ion Channel	Conductance S/cm^2^	Erev (mV)	Tissue	Reference
T- type Ca^2+^ channel	0.0002	51	Rat Bladder	Li et al., 2007 [56]
L- type Ca^2+^ channel	0.0004	51	Guinea pig Bladder	Sui et al., 2001 [17]
Voltage gated K^+^ channel–Kv1	0.006	─75	Mouse Bladder	Thorneloe et al., 2003 [61]
Voltage gated K^+^ channel-KCNQ	0.009	─75	Guinea pig bladder	Anderson et al., 2013 [64]
Calcium dependent K^+^ channel(BK)	0.024	─75	Murine bladder	Sprossmann, et al., 2009 [80]
Calcium dependent K^+^ channel(IK)	0.007	─75	Mouse intestinal smooth muscle	Vogalis et al., 1998 [81]
Calcium dependent K^+^ channel(SK)	0.01	─15	Guinea pig urinary bladder	Herrera et al., 2002 [40]
ATP dependent K^+^ channel	0.001	─21	Guinea pig urinary bladder	Bonev et al, 1993 [90]
Inward-rectifying channel	0.0001	─40	Rat bladder	Green et al.,1996 [91]

### Passive electrical properties of DSM cells

**Fig 7:**
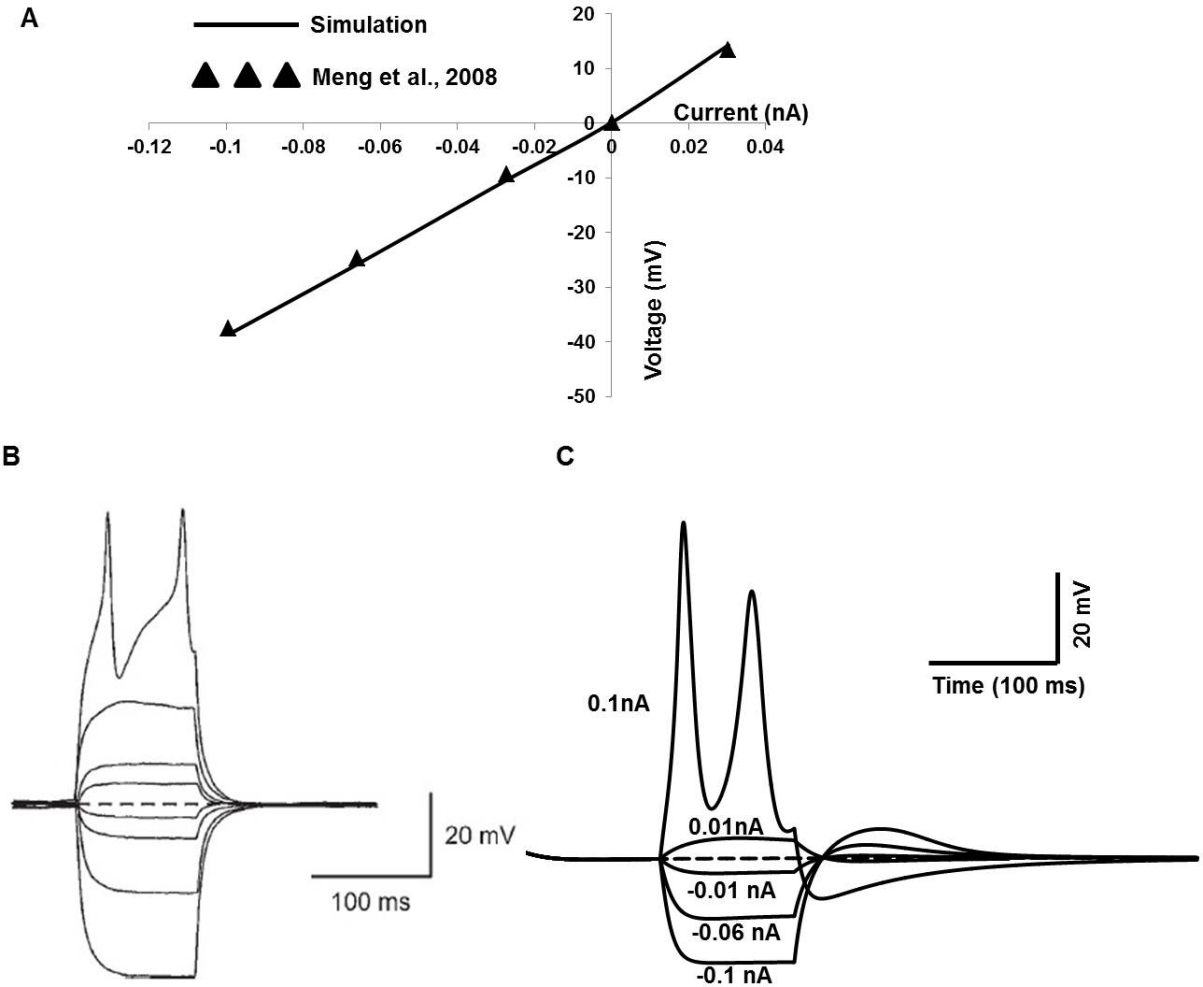
Passive and active membrane properties of DSM cell of the mouse bladder. **(**A) Simulated current–voltage relationship is shown in solid line against superimposed experimental data (filled triangles) [21]. (B) Experimental overlaid traces show the membrane potential changes from an active response cell in the mouse bladder induced by intracellular current injection of +0.1 to ─0.1 nA for 100 ms (with permission from [21]). The resting membrane potential is ─50 mV. (C) The simulated relation between the amplitude of injected currents (─0.1 to 0.1 nA for 100 ms) and resultant membrane potential.

Passive and active membrane properties of our single cell model are illustrated in Fig 7, compared to corresponding recordings from isolated mouse DSM cells taken from [21]. Fig 7A shows the simulated current–voltage relationship (solid line) obtained by a series of brief intracellular current injections (─0. 1 to 0.03 nA for 100 ms). Filled triangles represent adapted experimental data from Fig 5A in [21]. The relation between the amplitude of injected currents and resultant membrane potential changes is linear up to the threshold voltage. Two APs were fired (Fig 7C) by injecting a brief current pulse of 0.1 nA, which depolarizes the cell beyond approximately threshold voltage (─20 mV). At first glance, the major discrepancy from the experimental recording (Fig 5B, [21]) is that the experimentally evoked second AP exhibits a more prominent positive peak and concave foot step depolarization till the threshold voltage. The primary explanation for this discrepancy is that the experimental data are recorded in isolated bundle strip instead of isolated single cell.

### Whole cell membrane current and action potential

**Fig 8:**
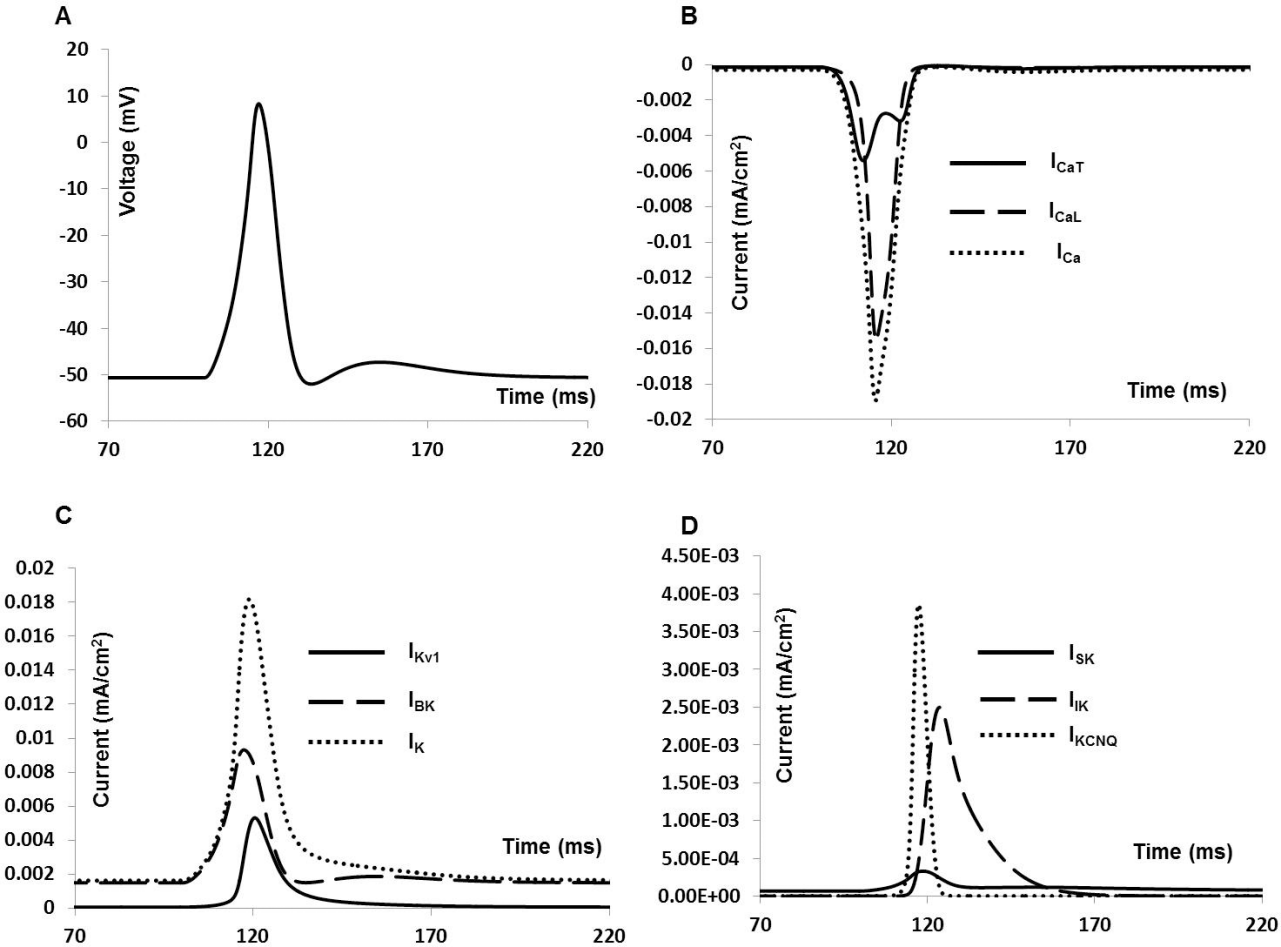
Current induced simulated AP and ionic currents. (A) Simulated AP. (B) Total inward current (dotted line), L- type Ca^2+^ channel current (dashed line) and T- type Ca^2+^ channel current (solid line). (C) Total outward current (dotted line), BK channel current (dashed line) and Kv1 channel current (solid line) (D) Outward current KCNQ channel current (dotted line), SK channel current (solid line) and IK channel current (dashed line).

DSM cells can fire different forms of spontaneous action potentials (sAPs) including a spike type AP, a pacemaker type AP, and APs with prominent after hyperpolarizations (AHPs) and after- depolarizations (ADPs). APs were induced in our model by applying an external stimulus, either as a brief rectangular pulse of current duration (0.5–50 ms) or as bi-exponential function with rising and falling time constants that mimic synaptic conductance. As the model cell possesses nine active conductances, a change in any individual active conductance can influence the cell’s electrical activities. Fig 8 illustrates the AP and associated ionic currents elicited by injecting 0.1 nA brief rectangular pulse for 10 ms. The voltage threshold is ≈ ─ 30 mV.

The AP in Fig 8A reached a maximum (V_***max***_) of +9.6 mV in 8 ms. The AP width (APW) measured at ─25 mV is about 35 ms, which agrees well with the experimental values [8, 20, 21]. The simulated AP also exhibits a hyperpolarization of amplitude 5 mV and lasting for 15 ms. Fig 8B shows total inward current (dotted line), L- type Ca^2+^channel current (dashed line) and T- type Ca^2+^ channel current (solid line) during the AP generation.

Total outward current (dotted line), BK channel current (dashed line) and Kv channel current (solid line) for the stimulated AP are shown in Fig 8C. Fig 8D displays KCNQ channel current (dotted line), SK channel current (solid line) and IK channel current (dashed line).

Intracellular electrophysiological recordings from mouse DSM cells reveal spontaneous depolarizations (SDs), distinguishable from sAPs by their amplitude (*<*40 mV) and insensitivity to the L-type Ca^2+^ channel blocker nifedipine [8]. It is well documented that SDs occur due to release of the purinergic neurotransmitter ATP [6, 7, 8].

**Fig 9:**
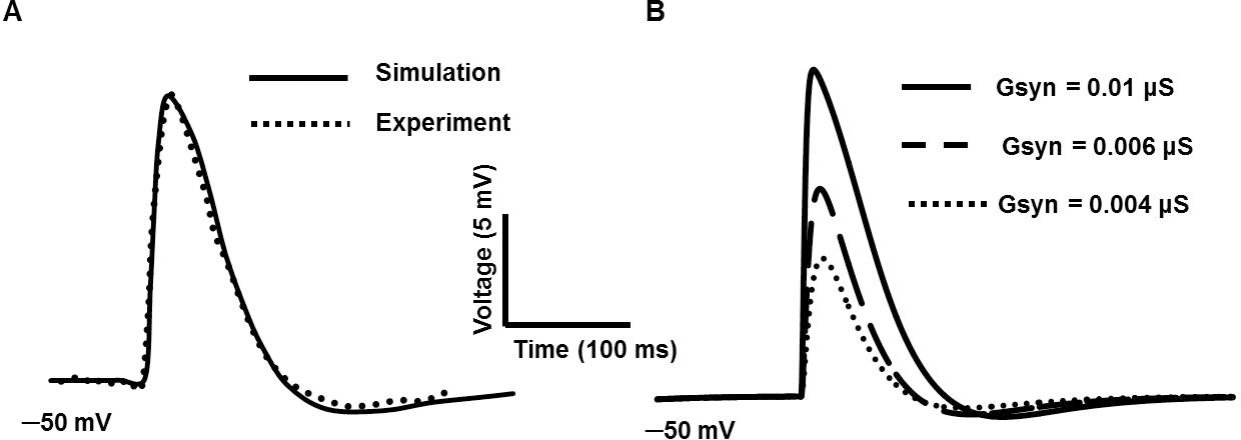
Simulated SDs with rapid rising phase and slower falling phase. (A) The solid line represents the simulated SD after setting the maximum conductance to 0.01 μS. The experimental data (dotted line) are plotted against the simulated one (solid line). (B) SDs with varying maximum conductance: 0.01 μS (thick solid line), 0.006 μS (long dashed line) and 0.004 μS (short dashed line).

Experimentally recorded SDs had mean peak amplitude of 5.9 mV with single exponential mean decay time constant of 49.6 ms [20]. Fig 9 shows a simulated SDs generated by using an exponential function with rapid rising phase and slower falling phase. The time constants for rise and fall are set to 5 ms and 50 ms respectively. In Fig 9A, the solid line represents the simulated SD after setting the value of maximum conductance to 0.01 μS. The data extracted from the experimental recording (dotted line, Fig 9A) in our lab are plotted against the solid line. Fig 9B illustrates the SDs of varying amplitudes produced by varying their conductances: 0.01 μS (thick solid line), 0.006 μS (long dashed line) and 0.004 μS (short dashed line). They closely resemble to experimental SDs reported in Fig 1C [20].

**Fig 10:**
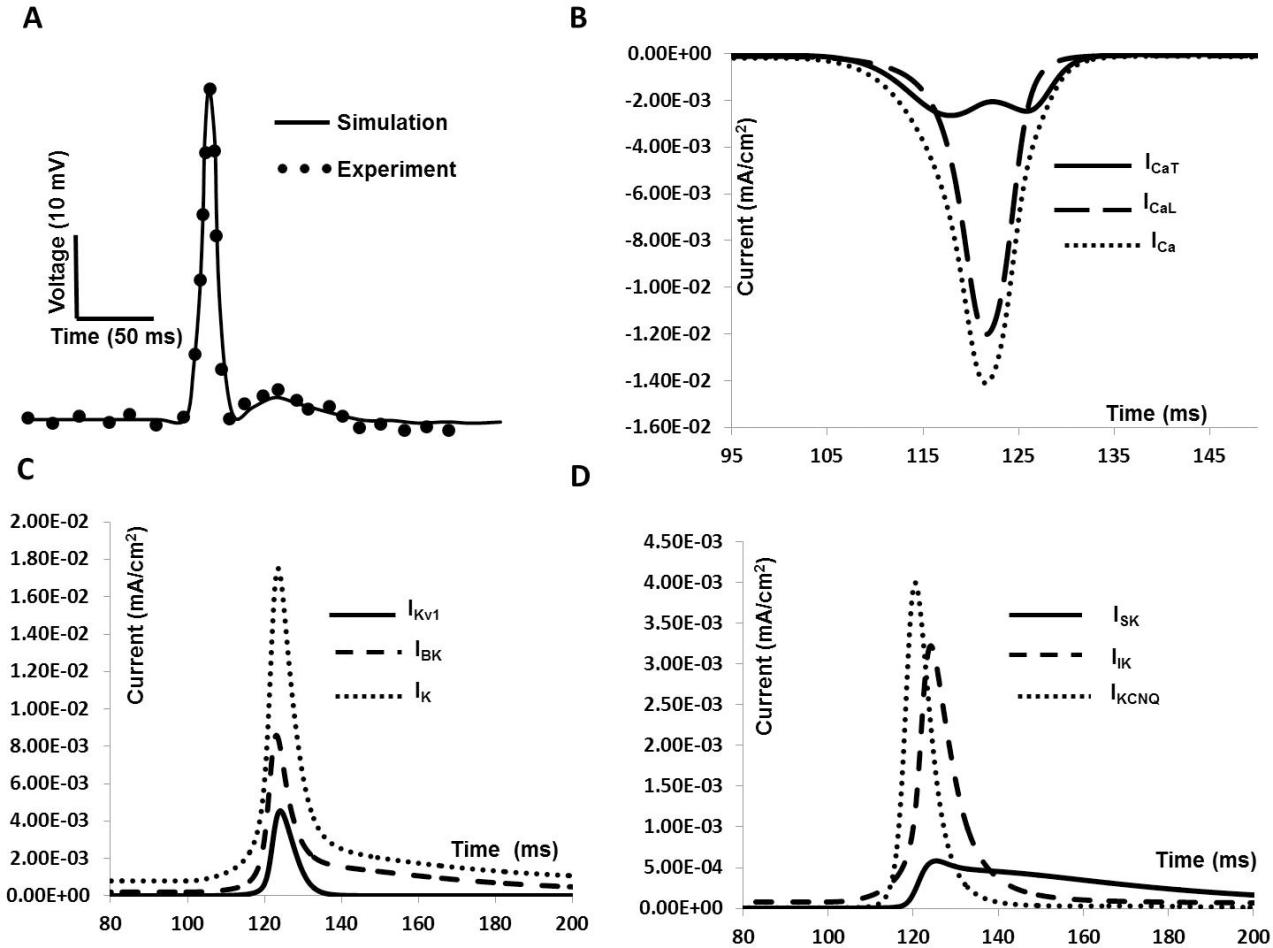
Synaptic input induced simulated AP and ionic currents. **(**A) Simulated AP. The superimposed filled circles represent data from experimental recordings (B) Total inward current (dotted line), L- type Ca^2+^ channel current (dashed line) and T- type Ca^2+^ channel current (solid line). (C) Total outward current (dotted line), BK channel current (dashed line) and Kv1 channel current (solid line) (D) KCNQ channel current (dotted line), SK channel current (solid line) and IK channel current (dashed line).

Following a synaptic input, our model is able to generate spike type APs with an ADP (Fig 10) riding on the repolarization phase. The conductance, rising phase and falling phase time constants for the synaptic conductance are set to 0.0095 μS, 15 ms and 25 ms respectively. Fig 10A shows the simulated AP with prominent ADP in repolarization phase. The experimental data are superimposed with filled circle. Fig 10B illustrates the total inward current (dotted line), L- type Ca^2+^ channel current (dashed line) and T- type Ca^2+^ channel current (solid line) during the AP generation. Total outward current (dotted line), BK channel current (dashed line) and Kv1 channel current (solid line) are shown in Fig 10C. Fig 10D shows KCNQ channel current (dotted line), SK channel current (solid line) and IK channel current (dashed line) during the repolarization period.

### Different types of action potential

**Fig 11:**
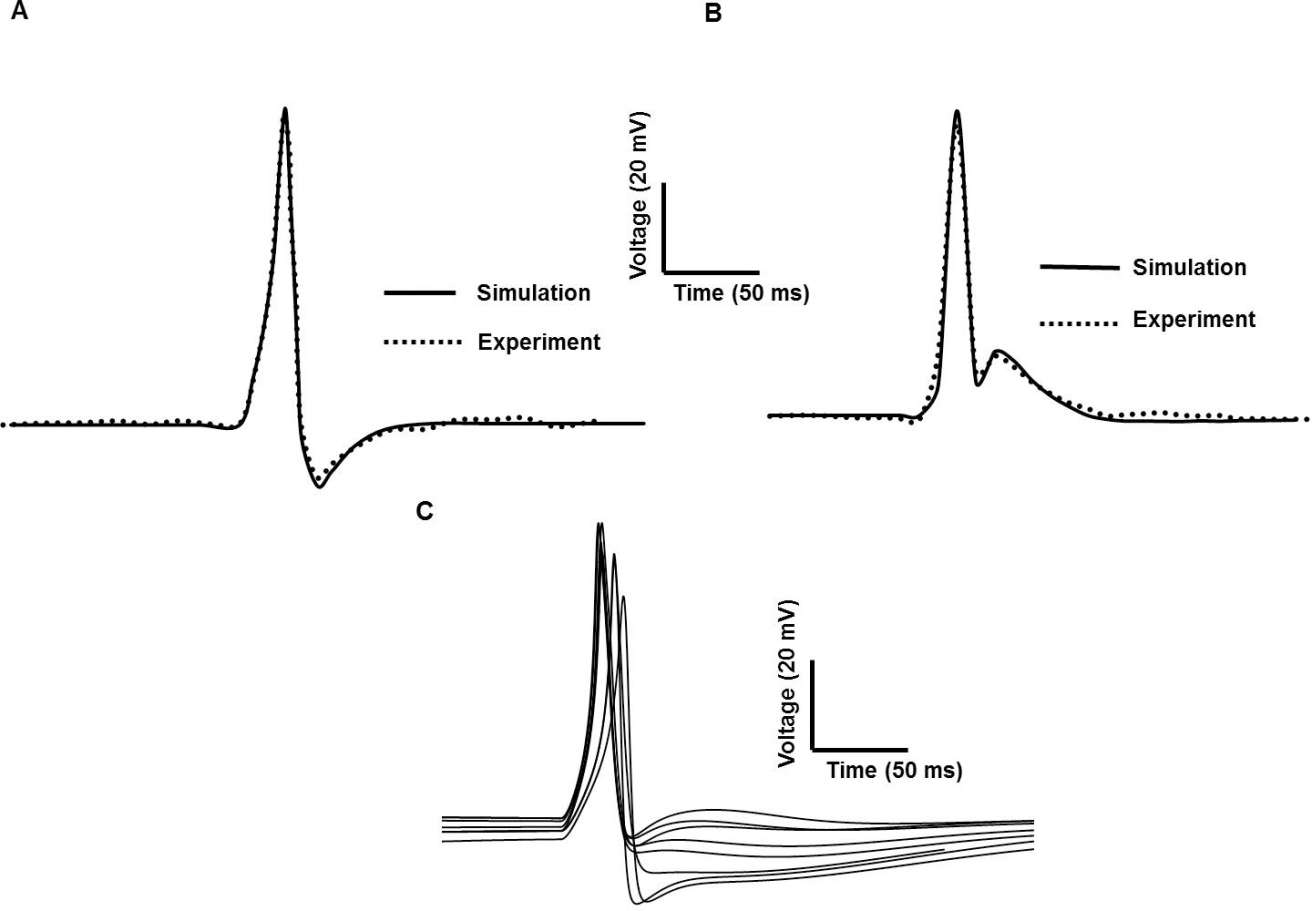
**Comparison of experimental & simulated spike-type APs of two different shapes produced by synaptic input with varying changes of conductance parameters (A and B). (**A) Conductances of 0.006 μS (B) Conductances of 0.02 μS. AP in (A) generates AHP while AP in (B) generates the prominent ADP. (C) APs produced by our model which corresponds to each of the experimental signals tabulated.

DSM cells can generate different forms of spike type APs including those exhibiting slow to fast AHP and ADP ([8]: Fig 3B). Most APs were preceded by slow depolarization but often had steeper foot-like depolarization ([20]: Fig 1B). One possible explanation for this AP shape variability is differences in the magnitudes of the intrinsic active ionic conductances. However, a second and equally plausible explanation is based upon synaptic input mechanisms. We varied the magnitude of synaptic conductance to study the underlying effects on AP shape without altering any other parameter. Fig 11 shows the spike type AP with ADP (Fig 11B) and AHP (Fig 11A) for conductances of 0.02 μS and 0.006 μS respectively. The unpublished experimental data are superimposed (dotted line) against the simulated APs (solid line). In Fig 11C we show superimposed a number of similar but differentiable AP shapes obtained by the same method, i.e. using a synaptic conductance as the depolarizing input. The ensemble of APs displayed here bear a close resemblance to the variety of shapes of spike-type APs that is recorded experimentally (see for example, Fig 1B, Ref 20). This suggests that one of the principal sources of AP shape variability in DSM cells may be variation in the underlying synaptic conductance, such that differing superpositions of the synaptic potential and the spike give rise to differing resultant AP shapes, as hypothesized previously [92]. Table 3 represents the comparison between simulated spike type APs and experimental observation in terms of RMP, AP amplitude (APA) and duration at 50% repolarization voltage level (APD_50_). These numerical matches indicate that our model is capable of accurately reproducing wave shapes previously reported experimentally, including their characteristic parameters. Fig 11C displayed the APs produced by our model which correspond to each of the experimental signals tabulated in Table 3.

**Table 3:** Comparison of spike type AP of mouse DSM cell: Experimental observation and model values.

Quantity	RMP (mV)		APA (mV)		APD_50_ (ms)	
	Exp	Model	Exp	Model	Exp	Model
Hayase et al., 2009 [20]	─43.5	-43.7	46	46.4	10	10.5
Young et al., 2008 [8]	─43	─43	53.4	54	38	37.4
Meng et al., 2008 [21]	─44	-43.7	47.8	47.5	23	22.7

### Role of Ca^2+^ channel in generating AP and total membrane current

**Fig 12:**
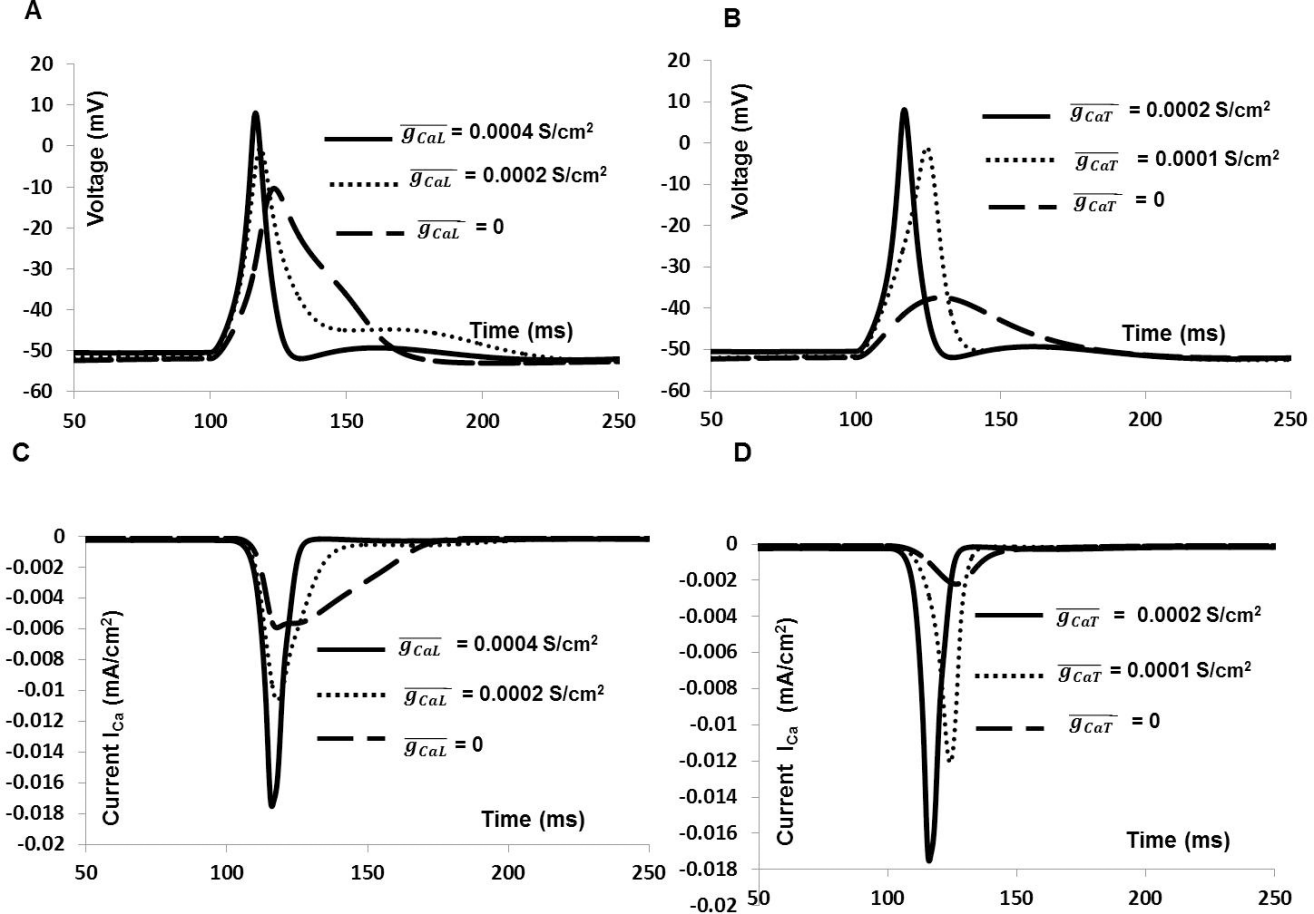
**Effects of inhibiting the inward currents I**_***CaT***_ **and I**_***CaL***_ **on the synaptic input based spikes (thick solid line, Fig 12A and Fig 12B) and total input current (thick solid line, Fig 12C and Fig 12D).** Fig 12A and Fig 12C show the spike and inward current with L- type Ca^2+^ channel conductance of 0.0004 S/cm^2^ (thick solid line), 0.0002 S/cm^2^ (dotted line) and 0 (dashed line). Fig 12B and Fig 12D show the spike and inward current with T- type Ca^2+^ channel conductance of 0.0002 S/cm^2^ (thick solid line), 0.0001 S/cm^2^ (dotted line) and 0 (dashed line).

To investigate the AP shape and whole cell membrane current further, each individual ion channel current was blocked by reducing the channel conductance. This explains how individual ionic current modulates certain phenomena observed in the mouse DSM cell AP. Fig 12 illustrates the effect of blocking I_CaL_ and I_CaT_ on AP (solid line, Fig 12A and Fig 12B) and total inward current (solid line, Fig 12C and Fig 12D) generated by synaptic input, which include significantly reduced V_***max***_ and inhibition of AP. Fig 12A and Fig 12C show the primary role of L- type Ca^2+^ channel in regulating shape of AP and total inward current. The peak amplitude of AP and total inward current are substantially reduced after blocking the L- type Ca^2+^ channel conductance by 50% (dotted line) and 100% (dashed line). Again, the significance roles of T—type Ca^2+^ channel conductance in eliciting AP and total inward current are reflected in Fig 12B and Fig 12D. Blocking I_CaT_ by 50% reduced the peak amplitude of AP (dotted line, Fig 12B) and inward current (dotted line, Fig 12D). However, 100% block of I_CaT_ results no AP (dashed line, Fig 12B) and inward current (dashed line, Fig 12D) in our model. The results show that both I_CaL_ and I_CaT_ play important roles in generating spike, although I_CaL_ is the major contributor to the total inward current.

### Role of K^*+*^ channel in shaping AP and generating total membrane current

**Fig 13:**
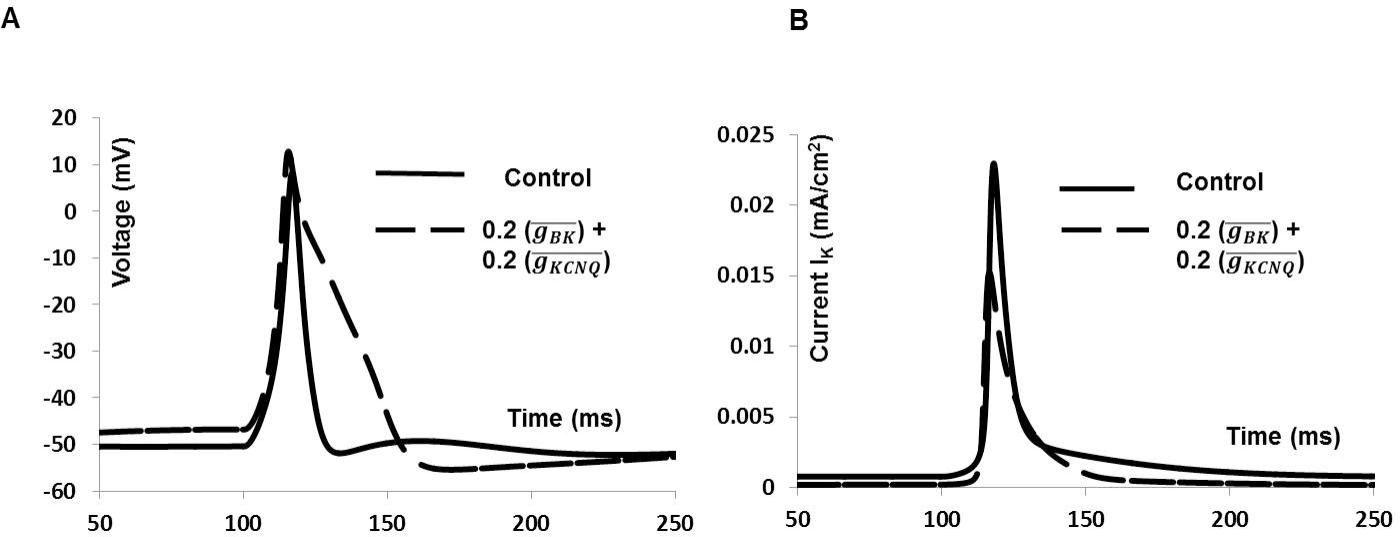
Effects of partially reducing conductance BK and KCNQ type K^+^ channels on the whole cell AP. (A) Synaptic input induced AP (solid line), BK and KCNQ type K^+^ channels 20% blocked AP (dashed line). (B) Total outward current for whole cell AP (solid line), BK and KCNQ type K^+^ channels partially blocked outward current (dashed line).

Two voltage gated K^+^ channels and three Ca^2*+*^ dependent K^+^ channels are incorporated in this model. Fig 13 shows the 20% blocking effect of BK and KCNQ type K^+^ channels on synaptic input induced AP and total outward current which includes depolarized RMP by 2 mV, increased V_***max***_ by 2.5 mV and prolonged AP duration (dashed line). This shows a good agreement with experimental finding (see ref 20, Fig 2D). Fig 13B shows the total outward current in control condition (solid line) and after reducing BK and KCNQ channels conductance (dashed line).

The results show that both BK and KCNQ channels play an important role in setting RMP, repolarization and BK channel is a major contributor to the total outward current.

**Fig 14:**
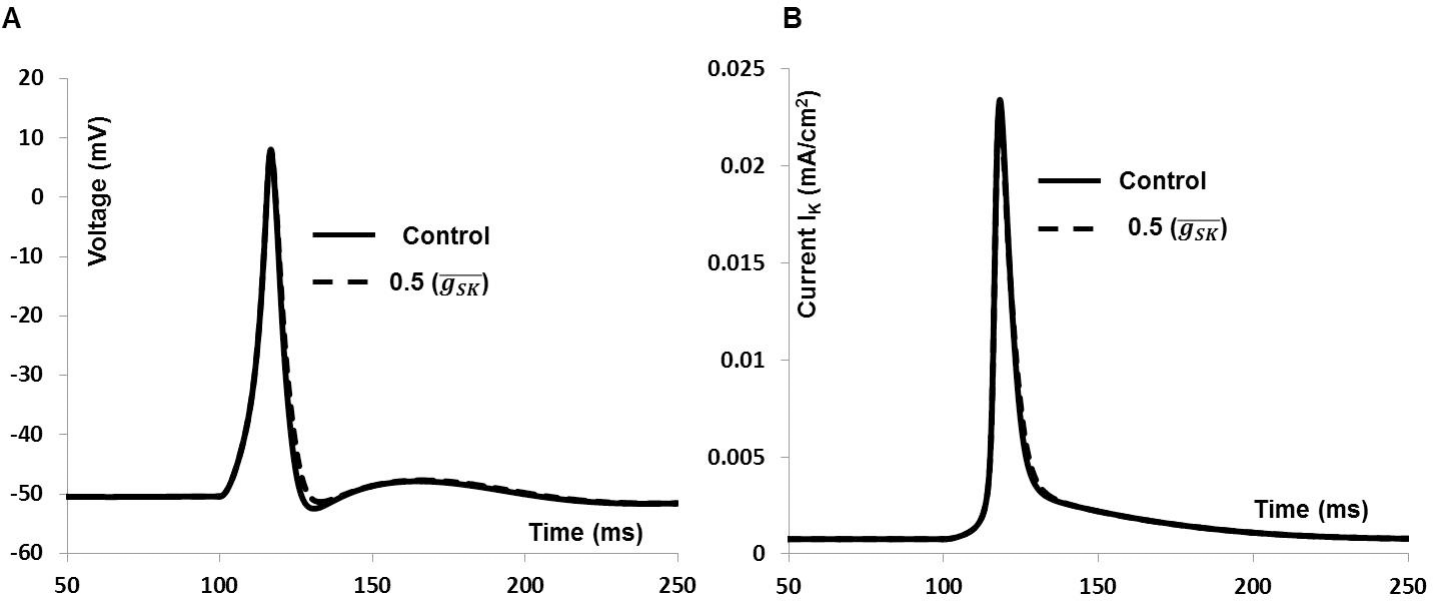
Effects of reducing (50%) conductance SK type K^+^ channels on the whole cell AP. **(**A) Synaptic input induced AP (solid line), SK type K^+^ channels blocked AP (dashed line). (B) Total outward current for whole cell AP (solid line), SK type K^+^ channels blocked outward current (dashed line).

We have also investigated effect of SK type K^+^ channels on synaptic input induced AP which includes a small change in AHP phase of AP. Fig 14A shows the AP in control condition (solid line) and after reducing SK channels conductance (dashed line). SK channels only modulate the AHP phase of the AP, which is consistent with experimental findings (see ref 20, Fig 3C). Fig 14B shows the total outward current in control condition (solid line) and after reducing SK channels conductance (dashed line). So, unlike BK and KCNQ channels, the SK channel does not contribute a significant amount towards total the outward current.

We have also validated the robustness of the model by altering selected conductances of the model’s ion channels by ±20% of their control value, taking such a percentage to represent variation within the physiological range. The Figure C, D and E in S1 File show the normalized variations in AP parameters with respect to normalized changes in L-type Ca^2+^, BK and K_V1_ conductances which are the major contributing conductances to the action potential. Here, the model ionic conductances are varied by up to ±20% of the control value in discrete steps. As can be observed, the model stays stable within the conductance ranges explored, the AP parameters varying only minimally in response, signifying robustness of our model to perturbations in ionic conductance. To further check model robustness, we also varied these conductances simultaneously to identical extents, up to ±20% of their control value. Our findings of this protocol on AP parameters are shown in Figure F in S1 File. The degree of change of these parameters is greater than that observed following similar changes made to individual ion channels. This is to be expected, owing to the inherent non-linear interactions between these conductances. However, importantly, the AP was still generated over the whole range (±20% of control) of simultaneous change in conductances explored, signifying robustness of the model to changes in these parameters.

### Simulation of simultaneous recordings of AP and cytosolic calcium [Ca^2+^]_i_

**Fig 15:**
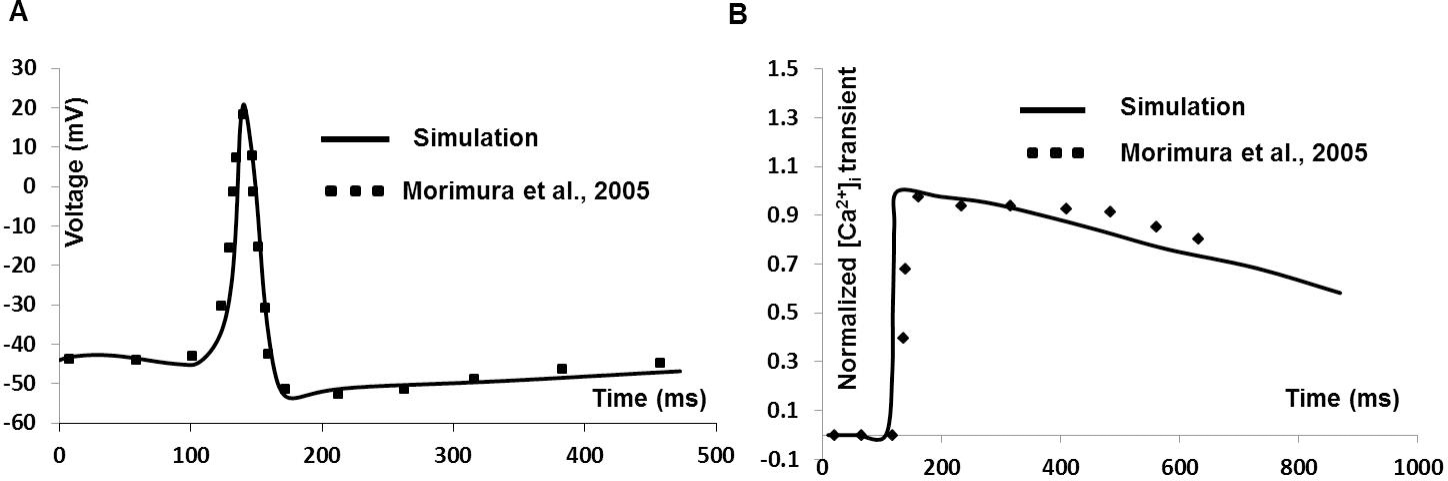
Simulated Ca^2+^ transient during AP. **(**A) Synaptic input induced simulated AP (Solid line) and superimposed experimental data (filled square). (B) Normalized simulated Ca^2+^ transient (Solid line) and extracted experimental data [93] (filled square).

We have investigated whether Ca^2***+***^ current via L-type Ca^2***+***^channel is responsible for firing of APs with fast upstroke generation. It is suggested that inhibition of L-type Ca^2***+***^ channel not only prevented AP generation, it also reduced the cytosolic Ca^2***+***^ transient. Fig 15 shows model predictions (solid line in Fig 15B) of [Ca^2+^]_i_ as a function of synaptic input induced AP (solid line in Fig 15A) next to extracted experimental data (filled square) from [93], where Ca^2+^ transient is recorded simultaneously during AP in mouse DSM cell. The RMP is set at –44 mV and Ca^2+^ transients are normalized before comparison. While the simulated cytosolic Ca^2***+***^ transient is not a precise fit to the experimental one, the concurrence is satisfactory.

According to Eq 8 (see Methods), the radius “r” and time constant τ_r_ of the shell influence the Ca^2+^ transient profile. In detrusor smooth muscle cells, the submembrane calcium transient occurs from a depth of 0.1 μm to a depth of 0.6 μm [94]. The Figure A in S1 File shows the modulating effect of radius “r” on the submembrane calcium transient profile. As expected from Eq 8, the Ca^2+^ transient amplitudes diminish as depth from the membrane increases. The Figure B in S1 File shows the relationship between radius “r” and time constant τ_r_ of the Ca^2+^ transient. It can be seen, as again expected from Eq 8, that τ_r_ bears an inverse relationship with radius “r”.

## Results III–spike propagation in a one-dimensional strand of DSM cells

**Fig 16:**
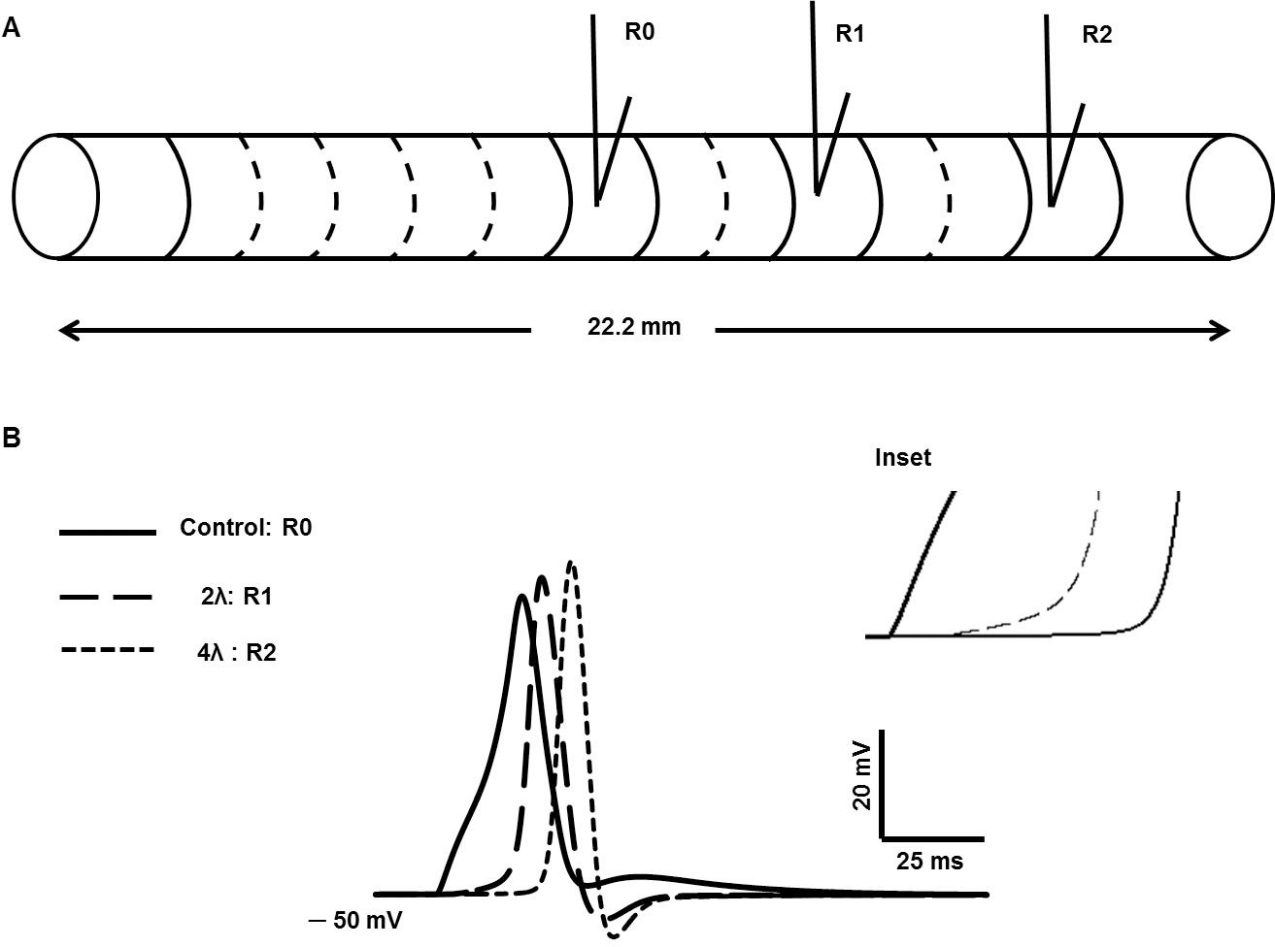
Simplified model of 1-D cable. (A) Unicellular compartmental 1-D cable with 111 segments. (B) Evoked AP at 11.1 mm (R0), propagated AP at 2λ (R1), and propagated AP at 4λ (R2). Inset shows the change in foot of propagated APs (long dashed line and short dashed line) due to spatial attenuation of the passive electrical activities.

To begin with, we set up the action potential in a single cell elongated to a large length (22.2 mm). We divided the cell into 111 interconnected compartments to behave as a continuous cable [25, 42], where each compartment was spatially isopotential. Fig 16A shows a simplified model of unicellular compartmental 1-D cable having the length of 22.2 mm and 111 compartments. The synaptic stimulus is injected at the midpoint of the cell, x = 11.1 mm and electrical activities are recorded at 11.1 mm (R0), 15.5 mm (R1) and 19.9 mm (R2) respectively.

The synaptic stimulus is injected at the midpoint of the cell, x = 11.1 mm and electrical activity is recorded at the point of stimulation, i.e., 11.1 mm (designated R0), at a distance of two length constants (2λ) from the point of stimulation (R1) and at a distance of four length constants (4λ) from the point of stimulation (R2), where the length constant (λ) for the DSM cell cable model was taken as 1.8 mm [42]. In Fig 16B, the AP at R0 (thick line) can be differentiated from the propagated APs (long dashed line and short dashed line) in terms of shape, width, peak value, and latency. The absence of the convex-upward foot and the ADP in the propagated APs is noticeable, while these components are prominent in the AP at R0. The peak amplitude of the propagated APs are also higher than the evoked control AP due to more charge dissipation to neighbouring segments in both directions for the evoked one. The inset displays in greater detail the transformation of the convex-upward foot of the AP at R0 into a concave-upward foot in the propagated APs, as expected from theory owing to the effect of cable properties. Given these findings, it can be hypothesized that some proportion of the varied AP shapes in DSM cells mentioned in the foregoing sections could be explained on the basis of whether, in experimental recordings, APs were recorded at or close to the locus of neurotransmitter action or at a distance from the locus (this distance itself being variable).

**Fig 17:**
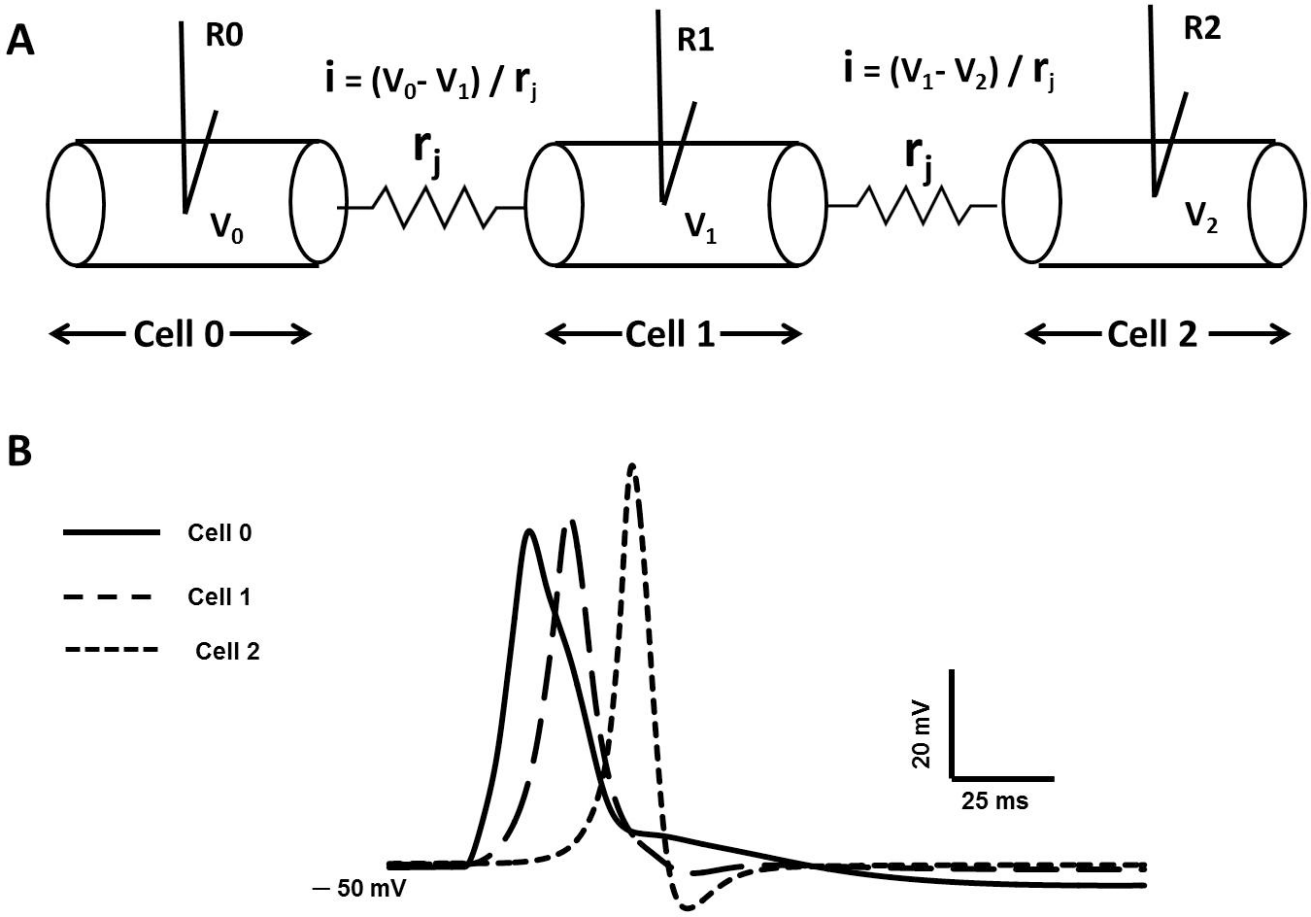
Gap junction implementation in multicellular 1-D cable. (A) three cells is connected by gap junction. (B) Evoked AP (V_0_) at cell 0 (R0), propagated AP (V_1_) at Cell 1 via one gap junction (R1), and propagated AP (V_2_) via two gap junctions (R2).

In syncytial tissues such as smooth muscle, the presence of gap junctions between cells underpins intercellular electrical communication [22, 52]. Variations in gap junctional coupling are a factor that can strongly affect propagation of electrical signals in a syncytium. We therefore extended our model to carry out a preliminary investigation of the effect of gap junction properties on propagated APs in DSM cells. Towards this end we first built a 3-cell model of electrically connected cells. In Fig 17A, r_j_ is the gap junction resistance among three multi-compartment (51 compartments) DSM cells (Cell 0, Cell 1 and Cell 2) and the junctions allow passage of localized currents by means of point processes mechanisms [25]. The V_0_, V_1_ and V_2_ are membrane potential of Cell 0, cell 1 and cell 2 with r_j_ value of 30 MΩ between the adjacent cells.

In Fig 17B, the thick solid line represents the evoked control AP recorded at R0 (Cell 0), the dashed line and thin line represents the propagated APs recorded at R1 (Cell 1) and R2 (Cell 2) via gap junctions. The shapes of propagated APs are also altered in both repolarizing and depolarizing phases. The foot of propagated APs is more concave with respect to that elicited at Cell 0 owing to spatial decay of the underlying synaptic input across the gap junction. The spatial decay is determined by the extent of gap junction coupling between the cells, thereby influencing the shape of the AP foot. Given these findings, it can further be hypothesized that some proportion of the different patterns of spikes recorded in DSM cells may arise from varying degrees of gap junction coupling among the cells.

## Discussion

We propose here a mathematical model of the cellular electrophysiology of mouse detrusor smooth muscle, including resting membrane properties and the action potential. The model successfully reproduces both passive and active electrical properties observed in intracellular recordings from individual DSM cells. The simulated results were validated against experimental recordings, some of these previously published and others obtained in our laboratory. Certain drug actions were also simulated towards model validation. We first developed the model for a single-compartment cell; this was then extended first to a long, multi-compartment, cable-like cell and next to a multicellular cable, in a preliminary attempt to investigate regenerative propagation of the DSM spike along a smooth muscle fibre.

A computational model for detrusor smooth muscle spikes has previously been reported [37]. In contrast to our model, which is biophysically explicit, the previous model is an implicit one. Although implicit models can be useful in certain analytic studies, several features of the model reported in the prior study, which comprised six ion channels, were at variance with physiologically measured ionic currents in the DSM, for instance in the following key respects. First, the properties of the ion channels employed were not validated against experimental data, such as ionic currents recorded under voltage clamp conditions and current–voltage curves derived from these. Many of the parameters used in the prior model were not referenced to experimental measurement and were therefore not constrained by biophysically known parameters. In contrast, we have generated DSM ionic currents using parameters drawn from published data (as cited in Methods and Results) and tuned these currents and their I-V curves so as to achieve the closest possible match to experimentally recorded signals. Second, the prior model is at odds with experimental findings as regards the complement of ion channels thought to be present in DSM cells. To the best of our knowledge, active voltage-gated Na^+^ and Ca^2+^ dependent Cl^-^ channels have not been documented for DSM cells of any species, yet the prior model has incorporated these channels in order to simulate the action potential. Conversely, while experimental studies provide substantial evidence for the presence in DSM cells of intermediate conductance Ca^2+^ -activated K^+^ channels, ATP-dependent K^+^ channels and inwardly-rectifying channels, these channels are not included in the model of Korogod et al., 2014 [37]. In endeavoring to build a physiologically more realistic model, constrained by available data, we have incorporated the latter channels but not the former. Third, the simulated spikes in the prior model were not tested against experimental spikes, whereas we have attempted to shape our spikes so as to achieve as close a match as possible to experimentally recorded ones, and have succeeded in doing so to a good degree of concordance.

In the light of the aforementioned points, therefore, we contend that our model is more biophysically detailed and constrained by experimental data to a greater degree than previous models, thereby constituting a formulation that lends itself better to heuristic predictions. We discuss in greater detail below the salient features of our construct, its performance against experimental data, and insights obtained from its use.

### Model features and performance

As a first step in our model development, passive electrical properties were simulated and validated against experimental recordings. The readouts from our model of the DSM myocyte’s passive properties, including resting membrane potential and the I-V relation of the cell in the subthreshold region, obtained by means of hyperpolarizing and depolarizing current injection, are consistent with experimental findings [21]. Altered values of resting membrane potential have been associated with DSM pathophysiology, for instance, the RMP of DSM cells has been reported to become continuously more positive in over activity [5, 21], making the RMP an important signal in its own right for analysis and investigation. It is noteworthy that the resting potential in our construct is an emergent property, arising from an interplay of several disparate conductances, including those for Na^+^, K^+^ and Ca^2+^, active to differing degrees at rest. Moreover, it is not predicated on the injection of a steady current in order to set the resting potential to the desired value, a procedure to which recourse is taken in some models [95, 96]. The “emergent” resting potential (~ –50 mV) of our model agrees well with values reported for DSM. This reposes confidence in the complement of ion channels we have included in our model and the default (resting) conductances assigned to them. By extension, it helps place confidence in the active signal—the action potential—engendered by the interplay of these channels in the suprathreshold region of operation. We discuss further the role of various ion channels in generating and altering the DSM resting potential in following section.

As a second step in model development and validation, we tested our simulated spikes against experimentally recorded ones, and found that we could mimic a range of AP shapes faithfully. Broadly speaking, two types of spike have been observed to occur spontaneously in DSM cells. One is the “pacemaker” type, which exhibits a long, ramp-like depolarization leading up to spike threshold and usually occurs in bursts. The other is the “spike” type, which has a briefer, convex-upward trajectory to threshold and usually occurs randomly in time, manifesting no evidence of bursting behaviour. The origins of the former type are unclear, whereas the latter are thought to arise following neurotransmitter activated depolarization in the smooth muscle cells, the neurotransmitter in question being ATP released from the parasympathetic motor innervation [6, 8, 63, 64]. Since there are considerably more data available on the spike-type action potential, we focused our attention on this category. In order to illustrate the difference between the pacemaker-type and the spike-type action potential, we have shown a typical pacemaker- type AP recorded from mouse DSM, in Figure G in S1 File.

We generated spike-type APs in our model by the use of two simulated inputs: (i) external current injection; (ii) synaptic potentials. As is evident from Figs 8A and 10A, the spike type APs triggered by either input closely match those observed in experimental recordings previously reported [20, 21]. They also matched spikes recorded intracellularly in our laboratory (as displayed in Fig 11A and 11B).

A particularly noteworthy feature of our model is that it is able to replicate not just one but a variety of action potential shapes (see Fig 11C). DSM is unusual in that spikes recorded from any one smooth muscle cell can exhibit several different shapes. We hypothesized that some of this variation may be accounted for by different degrees of superposition of the underlying purinergic synaptic potential, the dynamics of which can vary over a considerable range, and the intrinsic spike. It may be noted that synaptic potentials, termed spontaneous transient depolarizations [STDs] in the mouse DSM, do vary to the extent implemented in our model [8]. By altering the synaptic conductance over a realistic range, we were able successfully to simulate variation in STD configuration as well as a number of spike-type APs (featuring varying amplitudes of AHP and ADP) recorded from mouse DSM cells. This lends strength to the possibility that some of the shape variation observed in spike-type APs may be produced by differing degrees of superposition between the underlying synaptic potential and the evoked AP [92]. However other contributing mechanisms, such as variations in the complements of ion channels present in different smooth muscle cells, cannot be discounted.

A third feature of our model is that it incorporates a realistic profile of [Ca^2+^]_i_ in order to drive the Ca^2+^ dependent K^+^ channels present in DSM, which play a prominent part in shaping spike repolarization and the after-hyperpolarization. We derived the Ca^2+^ transient not from integration of individual intracellular Ca^2+^-handling components that comprise the Ca^2+^ signal (e.g. the Ca^2+^ release mechanisms of the sarcoplasmic reticulum membrane), but by implementing a forcing function that mimics its physiologically observed counterpart. This functions as a single-pool Ca^2+^ source that activates Ca^2+^ dependent K^+^ channels following influx of extracellular Ca^2+^ through voltage gated Ca^2+^ channels. A similar stratagem has been adopted in previous work [29] to simulate both the Ca^2+^ transient and the mechanical contraction that results from the Ca^2+^ transient in uterine smooth muscle. Although this procedure does not take into account individually the several factors that control intracellular Ca^2+^ dynamics, we verified that the Ca^2+^ transient thus generated bears a close agreement to that observed in experimental recordings [93]. It is therefore likely to satisfy the purpose for which we have employed it, i.e. controlling the mechanisms (e.g. Ca^2+^-activated K^+^ channels) that proximately govern the dynamics of the AP. In order to address the effects of any of the sub-components of Ca^2+^ dynamics on AP shape, a more detailed Ca^2+^ handling model is required, and we are currently working towards such a model in our laboratory.

An added attribute of our model is that it takes into account the temperature dependence of the ionic currents, based also upon data from experimental data recordings. Incorporation of temperature dependence is essential since ion channel properties such as steady-state activation and inactivation, the time constants of state parameters and the I-V relations, all exhibit their distinct temperature dependencies.

One of the salient properties of a canonical action potential is that it propagates regeneratively and without attenuation along the length of an excitable cell. Detrusor smooth muscle cells are thought to form a 3-D syncytium [41, 42]. We therefore sought to test our AP model further by seeing whether the simulated spike would propagate along a length of electrically continuous smooth muscle tissue. As a start toward this end, our model AP was incorporated into an elongated, one-dimensional cable model. Two types of 1-D cable model were explored: (i) a long unicellular cable (comprising 111 segments) without gap junctions and (ii) a multicellular 1-D cable, comprising 3 cells, each with 111 segments, with adjacent cells being electrically interconnected by gap junctions. Our simulations from these models were congruent with theoretical expectations. Moreover, while the AP at the site of its initiation displayed a convex-upward foot and a marked after-depolarization (ADP), the propagated AP exhibited the gradual waning of the convex foot as well as the ADP with respect to distance. This attribute too is predicted by cable theory, since the synaptic potential, which gives rise to both these components, is a non-regenerative signal and is expected to diminish in amplitude with distance while the regenerative AP propagates without decrement. We also established that the spatial characteristics of the decrement of the AP foot and the ADP matched those of “cable” or passive potentials produced by current injection. Beyond a distance of around four space constants, the components contributed by the STD were eliminated, which is as expected in an infinite cable.

### Contributions of ion channels to resting potential and action potentials

Both the resting potential and the profiles of action potentials are determined by a balance of the intrinsic ionic currents across the DSM cell membrane. It was therefore instructive to investigate, using our model, the contributions of the key ionic currents that modulate the shape of the AP and in turn determine features of critical physiological importance such as firing frequency, which eventually translates into strength of contraction. As we describe below, some of our findings support certain contentions advanced previously, while other findings question certain postulates and help resolve points of conflict in this domain.

There is broad agreement as to the role of T-type and L-type Ca^2+^ channel in initiating and regulating the spike. In our simulations, complete inhibition of I_CaT_ hyperpolarized the RMP, eliminated the AP and reduced DSM cell excitability. These findings are consistent with experimental results (Li et al., 2007 [56], Fig 8), where application of 200 μM NiCl_2_ (a T–type Ca^2+^ channel blocker) abolished evoked APs in rat DSM cells. Using our model we found that a two- fold increase in T-type Ca^2+^ channel conductance triggered the generation of APs in the absence of an input. This corroborates the central role of T-type Ca^2+^ channels proposed in the regulation of DSM cell excitability [56], wherein their activation triggers enhanced levels of spontaneous activity [17], chiefly by means of inducing a depolarization of resting potential that then leads to spontaneous APs being set off [57]. In regard to L-type Ca^2+^ channels, our simulations suggest that while inhibition of I_CaL_ suppresses or eliminates APs, the RMP is left unaffected (Fig 12). I_CaL_ is known to be the major contributor to the total inward current underlying the AP in DSM cells, as reported previously [8, 19], and our findings are consistent with this idea.

In contrast to the generally agreed roles of L-type and T-type Ca^2+^ channels, there exist several uncertainties in the realm of DSM electrophysiology as regards the relative contributions of individual K^+^ conductances to the various phases of the action potentials. In part, these uncertainties stem from the fact that any particular K^+^ channel blocker may influence the permeability of not just one but two or more of the K^+^ conductances present. According to Soder et al 2013 [84], Parajuli et al 2012 [71] and Li et al 2017 [39], elevated conductance of SK channels hyperpolarizes the RMP of DSM cells. Likewise, recent documents ascribed the hyperpolarization in murine DSM cells to SK channels, although the latter were postulated to be present in neighboring electrically connected interstitial cells, not in the DSM itself. In our simulations, blocking the SK conductance did not appreciably affect the total outward current during the AP (Fig 14A), nor did it alter the RMP subsequent to the after- hyperpolarization. Our findings therefore do not accord with the notion that SK channels hyperpolarize the RMP of DSM cells. Instead, our results point to a modulating effect of the SK conductance on the after-hyperpolarization of the AP. This is consistent with experimental findings reported [20, 40], from which it was concluded that SK channels modulate the spike frequency, but not the RMP.

Inhibition of voltage-gated and calcium-activated K^+^ currents, especially the KCNQ and BK currents respectively, produced marked effects in our simulations on DSM electrical characteristics. The RMP in both cases [KCNQ and BK channels] was depolarized by 1 mV and the APs generated had higher peak amplitudes and broader repolarization phases (Fig 13). These effects accord with those observed in experimental work, where voltage-dependent K^+^(KCNQ) and Ca^2+^-activated BK currents are reported to be dominant in modulating the RMP and shaping the repolarization phase of the AP in DSM cells [20, 59, 75]. A 50 per cent diminution in BK conductance depolarized the RMP to the threshold for L-type Ca^2+^ channel activation, consequently generating spontaneous APs without the application of an external stimulus. This result tallies with the observation of [97], where blocking the BK channels with iberiotoxin (IBTX) inhibited the whole cell outward K^+^ current, depolarized the resting potential, and increased the contractility of isolated human DSM strips.

Our findings suggest that BK channels have a number of roles to play in defining action potential shape and kinetics, being involved in the repolarization, the ADP, and the AHP of the spike; they also contribute to the maintenance of the RMP, these observations being consistent with those previously advanced [75, 97]. KCNQ or Kv2.1 channels also play a significant role in determining the characteristics of the after-hyperpolarization and the kinetics of the repolarization. We conclude that, unlike the BK and KCNQ conductances, the SK conductance may not contribute significantly towards the total outward current at the conductance levels employed in our model, which are derived directly from experimental reports.

### Model limitations and avenues for future work

Our model successfully replicated the spike type APs and underlying currents seen in mouse DSM cells. It is to be noted, however, that not all parameter values adopted in our model were obtained from mouse DSM, as quantitative electrophysiological data were not available in some instances for this tissue, obliging us to substitute parameters either from the DSM of other species or from other murine smooth muscles. For example, data for I_IKCa_ channels were adapted from mouse intestinal smooth muscle [81], some of the parameters (e.g. half-activation potential and slope of the activation parameter) being adjusted to fit the experimental data from mouse DSM.

Many parameter values that originated in other tissues needed to be tuned in order to give rise to action potentials that mimicked those recorded in murine DSM. Since we were able to achieve a reasonable match between experimental and simulated spikes, we feel that the tuned parameter values may provide acceptable “first-pass” estimates of the values that obtain physiologically.

A relatively simple model for calcium dynamics was built into our model. We used a forcing function in order to generate the Ca^2+^ transient required to activate Ca^2+^-dependent mechanisms that play a key part in shaping the DSM spike. A variety of Ca^2+^ release, uptake and buffering mechanisms, e.g. SER release channels, SERCA pumps and static and mobile buffers have been identified for certain smooth muscle and other excitable cells. A Ca^2+^ transient as generated by the operation of these multiple handling processes would render the model more complete, however it is unlikely to alter materially our findings and conclusions. This is because the [Ca^2+^]_i_-dependent channels that shape action potentials will to a good approximation activate, deactivate or inactivate identically so long as they experience the same final Ca^2+^ signal, regardless of whether it has been generated explicitly via individual release and uptake mechanisms, or implicitly via a forcing function. Moreover, it is not feasible at present to construct a biophysically detailed model of the Ca transient in DSM cells since precise values for the multiple parameters involved have not yet been delineated.

Our exploration of spike propagation by its incorporation into a 1-D model is a preliminary one. Because the smooth muscle of detrusor, akin to many other smooth muscles, forms a 3-D syncytium of cells, a biophysically realistic 3-D model is essential for a more physiologically realistic investigation. Appukuttan et al., 2015 [42] have reported a three-dimensional model for detrusor smooth muscle syncytium in the passive region of electrical functioning, incorporating gap junctional coupling between cells. This renders it possible in future work to insert the detailed action potential mechanisms reported here into the 3-D syncytium, allowing explorations of spike propagation in a more realistic topological setting, and we are currently addressing such questions. A further extension would be to incorporate models for contractile mechanisms triggered by the spikes reported here. Such multidimensional models will aid our understanding of DSM electrical and contractile function, providing windows of insight into the factors that govern excitability and contraction in both normal and unstable bladder, in turn shedding light on such phenomena as bladder overactivity and its underlying mechanisms.

## Supporting information

S1 Appendix Equations used in the model simulations. (PDF)

S1 File Supplementary figures. (PDF)

S1 Table Definitions of the equation symbols. (PDF)

S2 Table Constant parameters values used in model simulations. (PDF)
