# Supplementary material for: A biophysically constrained computational model of the action potential of mouse urinary bladder smooth muscle
Source: PLoS One. 2018 Jul 26;13(7):e0200712. doi: 10.1371/journal.pone.0200712 (PMC6061979; doi:10.1371/journal.pone.0200712)
Supplement: S1 Appendix — (PDF) [file pone.0200712.s001.pdf]

S1 Appendix: Equations used in the model simulations.

L-type Calcium current – I_{CaL}

$$I_{CaL} = \overline{g_{CaL}}\, n^2 hv\, hc(V - E_{CaL})$$

$$n_\infty = \frac{1}{1+\exp\left(\frac{-(V+9)}{8}\right)}$$

$$hv_\infty = \frac{1}{1+\exp\left(\frac{(V+30)}{13}\right)}$$

$$hc_\infty = \frac{ki}{ki+Cai}$$

$$\tau_n = \frac{0.001}{1+\ exp\left(\frac{-(V+22)}{308}\right)^1}$$

$$\tau_{hv} = 90 * (1 - \frac{1}{(1+exp\left(\frac{(V+14)}{45}\right)^1)\ (1+\exp(\frac{-(V+9.8)}{3.39}))})$$

$$\tau_{hc} = \ 20$$

T-type Calcium current – I_{CaT}

$$I_{CaT} = \overline{g_{CaT}}\, b^2 c(V - E_{CaT})$$

$$b_\infty = \frac{1}{1+\exp\left(\frac{-(V+32.1)}{6.9}\right)}$$

$$c_\infty = \frac{1}{1+\exp\left(\frac{(V+63.8)}{5.3}\right)}$$

$$\tau_b = \ 0.45 + \frac{3.9}{1+\left(\frac{(V+66)}{26}\right)^2}$$

$$\tau_c = (\ 150 - \frac{150}{(1+exp\left(\frac{(V-417.43)}{203.18}\right)^1)\ (1+\exp(\frac{-(V+61.11)}{8.07}))})$$

Voltage gated Potassium current – I_{Kv7} (KCNQ)

$$I_{KCNQ} = \overline{g_{KCNQ}}\, x^2 z^1 (V - E_K)$$

$$x_\infty = \frac{1}{1+\exp\left(\frac{-(V+7.1)}{15}\right)}$$

$$z_\infty = \frac{0.55}{1+\exp(\frac{(V+55)}{9})} + 0.45$$

$$\tau_x = (\frac{1}{1+\exp\left(\frac{(V+15)}{20}\right)})$$

$$\tau_z = 10 * (200 + \frac{10}{1+2\left(\frac{(V+56)}{120}\right)^5})$$

Voltage gated Potassium current – I_{Kv}

$$I_{Kv1} = \overline{g_{Kv1}}\, p^1 q (V - E_K)$$

$$p_\infty = \frac{1}{1+\exp\left(\frac{-(V+1.1)}{11}\right)}$$

$$q_\infty = \frac{1}{1+\exp(\frac{(V+58)}{15})}$$

$$\tau_p = (\frac{1}{1+\exp\left(\frac{(V+15)}{20}\right)})$$

$$\tau_q = 400 * (200 + \frac{10}{1+2*\left(\frac{(V+54.18)}{120}\right)^5})$$

Calcium dependent large potassium current – I_{BK}

$$I_{BK} = \overline{g_{BK}} * O * (V - E_K)$$

Common rate equations:

$$a = \exp\left(\frac{(38*v)}{380}\right), \qquad b = \exp\left(\frac{(17*v)}{330}\right)$$

K_{on} = 345, K_{coff} = 25, K_{ooff} = 25

O = O1 + O2 + O3 + O4

Rate equations for voltage dependent transitions:

K_{C0O0} = 0.02162 * a, K_{C1O1} = 0.000869 * a, K_{C2O2} = 0.0000281 * a, K_{C3O3} = 0.000781 * a, K_{C4O4} = 0.044324 * a,

K_{O0C0} = 318.1084 * b, K_{O1C1} = 144. 1736 * b, K_{O2C2} = 32.6594 * b, K_{O3C3}= 0.095312 * b, K_{O4C4}= 0.000106 * b * cai

Rate equations for calcium dependent transitions:

K_{C0C1}= 4 * K_{on} * cai, K_{C1C2}= 3* K_{on} * cai, K_{C2C3}= 2 * K_{on} * cai, K_{C3C4}= K_{on} * cai

K_{C4C3}= 4 * K_{coff} * cai, K_{C3C2}= 3 * K_{coff} * cai, K_{C2C1}= 2 * K_{coff} * cai, K_{C1C0}= K_{coff} * cai

K_{O0O1}= 4 * K_{on} * cai, K_{O1O2}= 3 * K_{on} * cai, K_{O2O3}= 2 * K_{on} * cai, K_{O3O4}= K_{on} * cai

K_{O4O3}= 4 * K_{ooff} * cai, K_{O3O2}= 3 * K_{ooff} * cai, K_{O2O1}= 2 * K_{ooff} * cai, K_{O1O0} = K_{ooff} * cai

Calcium dependent intermediate potassium current – I_{IK}

$$I_{IK} = \overline{g_{IK}} * l^2 * k * (V - E_K)$$

$$l_\infty = \frac{0.27}{1 + \exp\left(\frac{(li - v)}{15}\right)}$$

$$li = -190 + (37 * exp^{(ci*50)}) + (96 * exp^{(-27*ci)})$$

$$\tau_l = 15 * \left(\left(\frac{20}{1+\exp\left(\frac{(V+20.52)}{35}\right)}\right)\right)$$

$$k_\infty = \frac{0.32}{1 + \exp\left(\frac{(66 + v)}{8}\right)}$$

$$\tau_k = 45 * (1 - \frac{1}{(1 + \exp\left(\frac{(v + 13.9629)}{45.3782}\right)) * (1 + \exp\frac{-(v + 9.49866)}{3.3945})})$$

Calcium dependent small potassium current – I_{SK}

$$I_{SK} = \overline{g_{SK}}\, r^2 * (V - E_K)$$

$$r_\infty = 1.3 \exp 4 * (\frac{cai}{1.1})^4 / (1.3 \exp 4 * (\frac{cai}{1.1})^4 + 0.06^1$$

$$\tau_r = \frac{0.2}{1.3 \exp 4 * (\frac{cai}{1.1})^4 + 0.06^1}$$

Inwardly-rectifying channel - I_h

$$I_h = \overline{g_h}\, s^2 (V - E_h)$$

$$s_\infty = \frac{1}{1+\exp\left(\frac{(V-85)}{16}\right)}$$

$$\tau_s = 5 * \left(\frac{15}{(1+\exp\left(\frac{(49.042-V)}{10}\right))}\right)$$

ATP-sensitive K⁺ channel (K_{ATP})

$$I_{katp} = \overline{g_{KATP}} * t * (V_m - E_{rev}\ )$$

$$t_{\infty} = {}^{1}\Big/_{\left(1 + \left(\frac{ATPh}{ATPi}\right)^{n}\right)}$$

$$\tau_t = 1 + \frac{1}{(1.37 + \ (\mathrm{atpi} * \mathrm{exp}\left(\frac{(1)}{187}\right))}$$
