# Supplementary material for: A biophysically constrained computational model of the action potential of mouse urinary bladder smooth muscle
Source: PLoS One. 2018 Jul 26;13(7):e0200712. doi: 10.1371/journal.pone.0200712 (PMC6061979; doi:10.1371/journal.pone.0200712)
Supplement: S1 File — (PDF) [file pone.0200712.s002.pdf]

S1 File: Supplementary figures

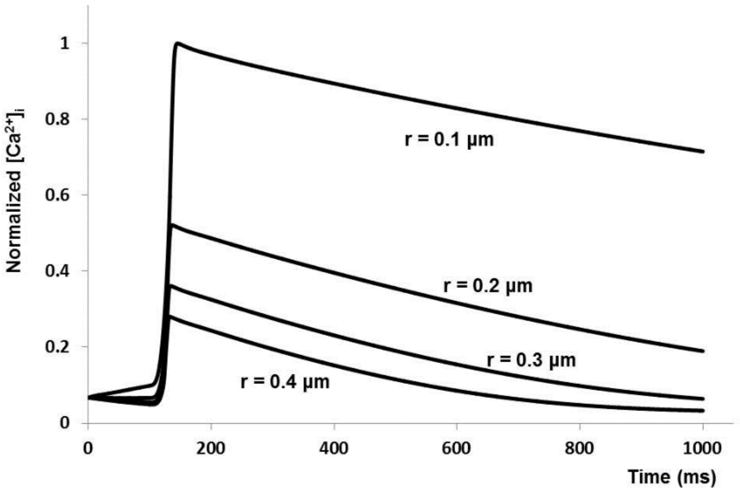

Figure A. $[Ca^{2+}]_i$ profile with depth (r) values of 0.1 µm, 0.2 µm, 0.3 µm and 0.4 µm. Since the Ca^{2+} profile at the BK channel is the one that occurs in the submembrane shell, the Ca^{2+} profile at a depth of 0.1 µm (r = 0.1 µm) represents the Ca^{2+} profile at the BK channel.

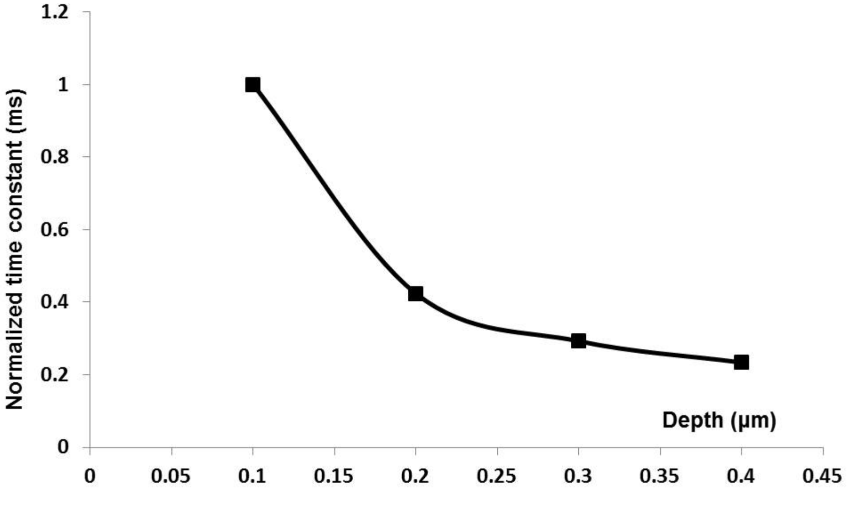

Figure B. Relationship between and geometry (depth) of shell and time constant (τ_r) of $[Ca^{2+}]_i$ profile. It is shown that the τ_r bears an inverse relationship with radius "r", as expected from equation 8.

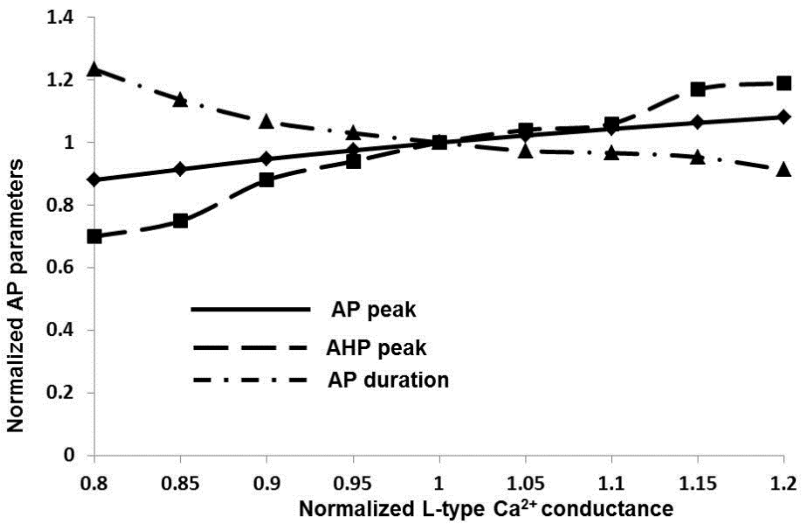

Figure C. Robustness analysis with varying L- type Ca^{2+} conductance. The model L- type Ca^{2+} conductance is varied by up to ± 20 % of the control value in discrete steps. The changes in AP parameters are shown for AP peak, AHP peak and AP duration respectively. Note that each AP parameter varies at most by up to + 20% and ─ 30%, indicating stability of the AP in the face of L- type Ca^{2+} conductance range.

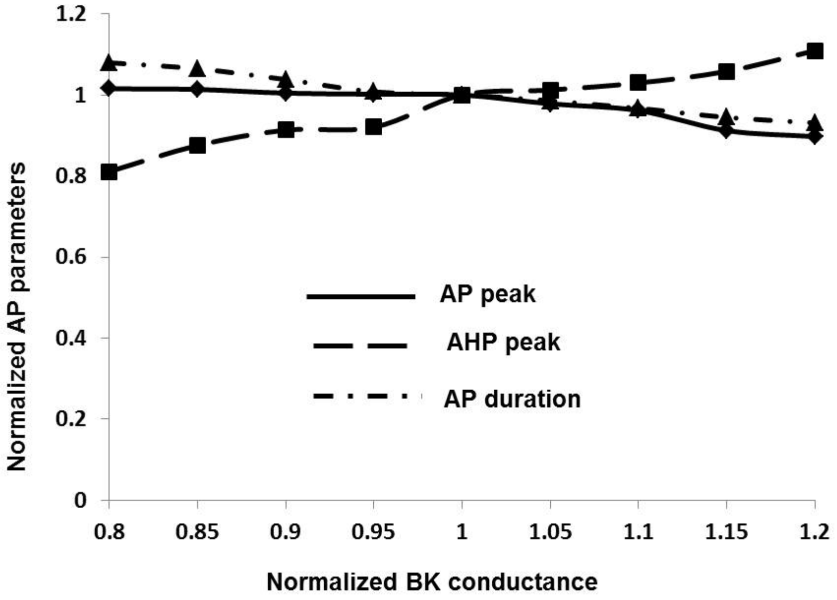

Figure D. Robustness analysis with varying BK conductance. The model BK conductance is varied by up to ± 20 % of the control value in discrete steps. The changes in AP parameters are shown for AP peak, AHP peak and AP duration respectively. Note that each AP parameter varies at most by up to + 10% and ─ 20%, indicating stability of the AP in the face of BK conductance range.

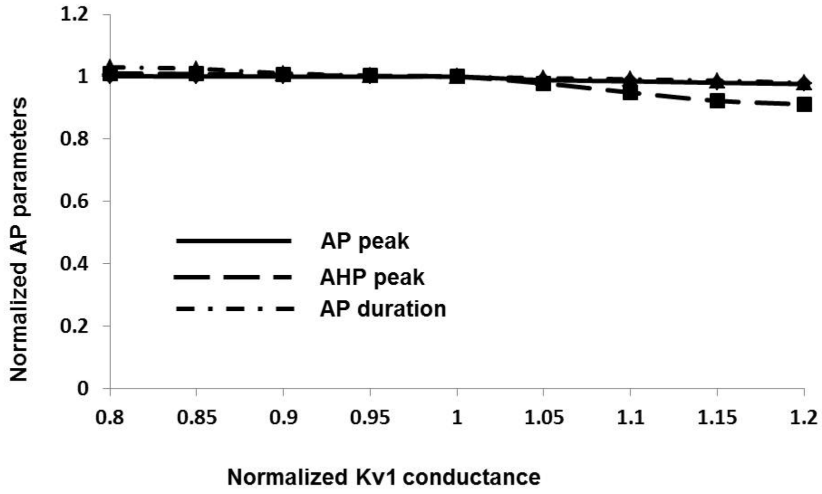

Figure E. Robustness analysis with varying Kv1 conductance. The model Kv1 conductance is varied by up to ± 20 % of the control value in discrete steps. The changes in AP parameters are shown for AP peak, AHP peak and AP duration respectively. Note that each AP parameter varies at most by up to only ± 5%, indicating stability of the AP in the face of Kv1 conductance range.

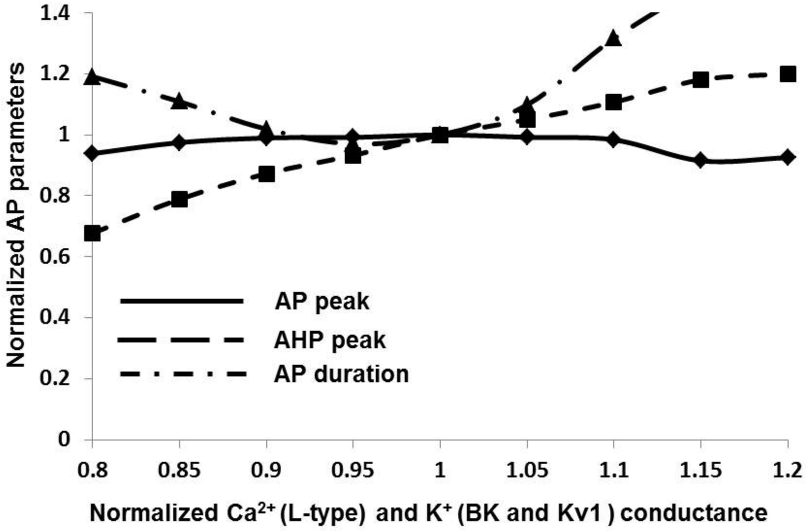

Figure F. Robustness analysis with varying model ionic (L- type Ca^{2+}, BK and Kv1 type K^{+}) conductances simultaneously. The model L- type Ca^{2+} conductance, BK and Kv1 type K^{+} conductances are varied simultaneously by up to ± 20 % of the control value in discrete steps. The changes in AP parameters are shown for AP peak, AHP peak and AP duration respectively. Note that each AP parameter varies at most by up to + 40% and ─ 30%, indicating stability of the AP in the face of simultaneous variation in L-type Ca^{2+}, BK and Kv1 conductances. The degree of change of these parameters is greater than that observed following similar changes

made to individual ion channels. This is to be expected, owing to the inherent non-linear interactions between these conductances.

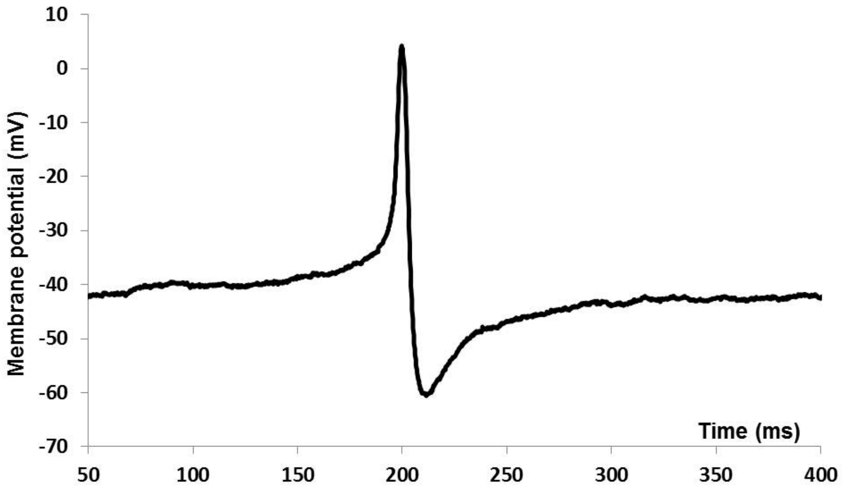

Figure G. Pacemaker type AP in mouse DSM Cell (recording by M Padmakumar and KL Brain, unpublished work). Note the prolonged ramp like depolarization leading to the spike, and the large AHP, which distinguish this type of AP from the spike type AP.
