# Supplementary material for: A biophysically constrained computational model of the action potential of mouse urinary bladder smooth muscle
Source: PLoS One. 2018 Jul 26;13(7):e0200712. doi: 10.1371/journal.pone.0200712 (PMC6061979; doi:10.1371/journal.pone.0200712)
Supplement: S1 Table — (PDF) [file pone.0200712.s003.pdf]

S1 Table: Definitions of the equation symbols

Symbols	Meaning
v	membrane potential
$V_{0.5}$	half (in)activation constant
V_h	holding potential
Q_{10}	Temperature coefficient
Ca^{2+}	calcium ion
Na^+	sodium ion
K^+	potassium ion
Cl^-	chloride ion
$[Ca^{2+}]_i$	intracellular calcium
$[Ca^{2+}]_o$	extracellular calcium
E_{rev}	reversal potential
E_{CaL}	reversal potential of I_{CaL}
E_{CaT}	reversal potential of I_{CaT}
E_h	reversal potential of I_h
E_K	reversal potential of potassium currents
I	current
I_{CaL}	L-type Ca^{2+} current
I_{CaT}	T-type Ca^{2+} current
I_{KCNQ}	Voltage-gated K^+ current (KCNQ)
I_{Kv1}	Voltage-gated K^+ current (Kv1)
I_{BK}	Ca^{2+}-activated K^+ current (BK)
I_{IK}	Ca^{2+}-activated K^+ current (IK)
I_{SK}	Ca^{2+}-activated K^+ current (SK)
I_h	Inward rectifying current
I_{KATP}	ATP activated K^+ current
$\overline{g_{CaL}}$	maximum conductance of I_{CaL}
$\overline{g_{CaT}}$	maximum conductance of I_{CaT}
$\overline{g_{KCNQ}}$	maximum conductance of I_{Kv7}
$\overline{g_{Kv1}}$	maximum conductance of I_{Kv1}
$\overline{g_{BK}}$	maximum conductance of I_{BK}
$\overline{g_{IK}}$	maximum conductance of I_{IK}
$\overline{g_{SK}}$	maximum conductance of I_{SK}
$\overline{g_h}$	maximum conductance of I_h
$\overline{g_{KATP}}$	maximum conductance of I_{KATP}
n	Activation gate of I_{CaL}
hv	Voltage gated inactivation gate of I_{CaL}
hc	Ca^{2+} gated inactivation gate of I_{CaL}
b	Activation gate of I_{CaT}
c	Inactivation gate of I_{CaT}
x	Activation gate of I_{KCNQ}
z	Inactivation gate of I_{KCNQ}
p	Activation gate of I_{Kv1}
q	Inactivation gate of I_{Kv1}

l	Activation gate of I_{IK}
k	Inactivation gate of I_{IK}
o	Opening state of I_{BK}
r	Ca^{2+} activated gate of I_{SK}
s	Activation gate of I_h
t	Activation gate of I_{KATP}
ATP_h	disassociation constant
ATP_i	intracellular ATP concentration
