# Supplementary material for: A biophysically constrained computational model of the action potential of mouse urinary bladder smooth muscle
Source: PLoS One. 2018 Jul 26;13(7):e0200712. doi: 10.1371/journal.pone.0200712 (PMC6061979; doi:10.1371/journal.pone.0200712)
Supplement: S2 Table — (PDF) [file pone.0200712.s004.pdf]

S2 Table: Constant parameter values used in model simulations.

Parameters	Values
$[Ca^{2+}]_i$	150 nM
E_{KATP}	$-$21 mV
E_{CaL}	51mV
E_{CaT}	51mV
E_K	$-$75 mV
E_h	$-$40 mV
ATP_h	0.006 mM
ATP_i	0.1 mM
$\overline{g_{CaL}}$	0.0006 S/cm^2
$\overline{g_{CaT}}$	0.0003 S/cm^2
$\overline{g_{KCNQ}}$	0.0001 S/cm^2
$\overline{g_{Kv1}}$	0.0008 S/cm^2
$\overline{g_{BK}}$	0.00015 S/cm^2
$\overline{g_{IK}}$	0.0001 S/cm^2
$\overline{g_{SK}}$	0.0001 S/cm^2
$\overline{g_h}$	0.0002 S/cm^2
$\overline{g_{KATP}}$	0.0004 S/cm^2
d	0.41 μm
τ_r	1800 ms
